# High-Throughput Single-Molecule Sensors: How Can the Signals Be Analyzed in Real Time for Achieving Real-Time Continuous Biosensing?

**DOI:** 10.1021/acssensors.3c00245

**Published:** 2023-05-22

**Authors:** Max H. Bergkamp, Sebastian Cajigas, Leo J. van IJzendoorn, Menno W.J. Prins

**Affiliations:** † Department of Biomedical Engineering, Eindhoven University of Technology, Eindhoven 5612 AE, The Netherlands; ‡ Department of Applied Physics and Science Education, Eindhoven University of Technology, Eindhoven 5612 AE, The Netherlands; § Institute for Complex Molecular Systems (ICMS), Eindhoven University of Technology, Eindhoven 5612 AE, The Netherlands; ∥ Helia Biomonitoring, Eindhoven 5612 AR, The Netherlands

**Keywords:** real-time, continuous biosensing, single-molecule sensors, high-throughput, signal processing, data analysis

## Abstract

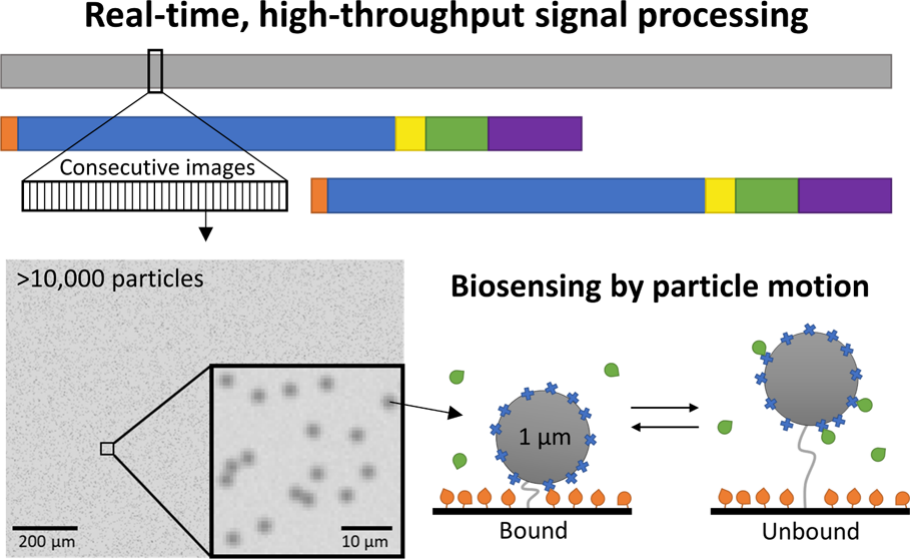

Single-molecule sensors collect statistics of single-molecule interactions, and the resulting data can be used to determine concentrations of analyte molecules. The assays are generally end-point assays and are not designed for continuous biosensing. For continuous biosensing, a single-molecule sensor needs to be reversible, and the signals should be analyzed in real time in order to continuously report output signals, with a well-controlled time delay and measurement precision. Here, we describe a signal processing architecture for real-time continuous biosensing based on high-throughput single-molecule sensors. The key aspect of the architecture is the parallel computation of multiple measurement blocks that enables continuous measurements over an endless time span. Continuous biosensing is demonstrated for a single-molecule sensor with 10,000 individual particles that are tracked as a function of time. The continuous analysis includes particle identification, particle tracking, drift correction, and detection of the discrete timepoints where individual particles switch between bound and unbound states, yielding state transition statistics that relate to the analyte concentration in solution. The continuous real-time sensing and computation were studied for a reversible cortisol competitive immunosensor, showing how the precision and time delay of cortisol monitoring are controlled by the number of analyzed particles and the size of the measurement blocks. Finally, we discuss how the presented signal processing architecture can be applied to various single-molecule measurement methods, allowing these to be developed into continuous biosensors.

## Introduction

Biosensing technologies for the continuous monitoring of biomolecules are set to become of great value in areas such as wearable sensors,^1,2^ healthcare,^3−5^ and industrial processing.^6,7^ The most well-known sensor for biomolecular monitoring is the subcutaneous continuous glucose sensor. The sensor monitors the concentration of glucose in the skin and helps diabetic patients to continuously optimize their insulin treatment. Unfortunately, the basic principles underlying present-day glucose sensors cannot be ported to measuring other molecules such as hormones, drugs, proteins, and nucleic acids because these analytes are present at much lower concentrations, ranging from micromolar to nanomolar and picomolar. Thus, more sensitive technologies are needed for realizing continuous real-time biomolecular sensing for a variety of applications.

A strategy to increase sensitivity is by harnessing single-molecule measurement principles.^8−12^ Examples are the single-molecule enzyme-linked immunosorbent assay (digital ELISA),^13,14^ single-molecule fluorescence,^15,16^ single-molecule plasmonics,^17,18^ and single-molecule nanopores.^19,20^ However, it is difficult to enable continuous real-time biosensing because (1) the underlying sensing principle needs to be reversible, such that increases as well as decreases of analyte concentration can be continuously followed as a function of time; (2) time-dependent signals need to be available in high throughput, such that sufficient single-molecule statistics can be collected in a limited amount of time; and (3) a signal processing methodology should be developed that can continuously analyze the data in real time and that is able to control the trade-off between analysis time and the required analytical precision.

A single-molecule measurement methodology that has been designed for continuous monitoring is biosensing by particle motion (BPM).^21−26^ The method relies on tracking the motion of individual biofunctionalized particles that interact with a biofunctionalized substrate. The particles switch between bound and unbound states due to reversible single-molecule interactions influenced by the presence of analyte molecules, so that the switching rate depends on the analyte concentration in solution. However, in previous works, the BPM sensing data was analyzed only after the completion of the assay experiment rather than in real time during the assay experiment. Therefore, a signal processing architecture needs to be developed that enables the continuous analysis of sensing data in real time, including the possibility to optimize the trade-off between analytical precision and time delay of the biosensor.

In this paper, we present a high-throughput signal processing architecture for real-time continuous biosensing based on single-molecule interactions, demonstrated on a BPM biosensor with more than 10,000 particles. The real-time biosensing is validated using simulated data and tested in an experiment with real-time cortisol sensing. The sensor used in this research is a reversible competitive BPM sensor that can continuously monitor increases as well as decreases in cortisol concentrations,^26^ which has been a limitation in earlier work.^27−30^ The results show how the trade-off between analytical precision and time delay of continuous biosensing can be controlled. Finally, we discuss how the developed signal processing architecture can be applied to other single-molecule measurement principles.

## Methods

### Definitions

The following definitions will be used:

Continuous monitoring: refers to a process and technology to continuously collect measurement data from a system of interest with a well-defined frequency and time delay. The frequency is the rate at which measurement data are reported. The time delay is the difference between the time at which a measurement result is reported and the time at which the system of interest was really in that condition.

Continuous biosensing: refers to continuous monitoring using a biosensor. The time delay is the difference between the time at which a concentration measurement is reported and the time at which the system of interest was really in that concentration condition. Continuous biosensing requires a sensing principle that responds in a reversible manner to interactions with analyte molecules in order to allow monitoring of increases as well as decreases of the analyte concentration.

Real-time continuous biosensing, or real-time biosensing in short: refers to continuous biosensing with a time delay that is short with respect to the timescales of typical concentration fluctuations in the system of interest.

Real-time signal processing: refers to signal processing with time characteristics that enable real-time continuous biosensing.

### Basic Considerations

**Figure 1:**
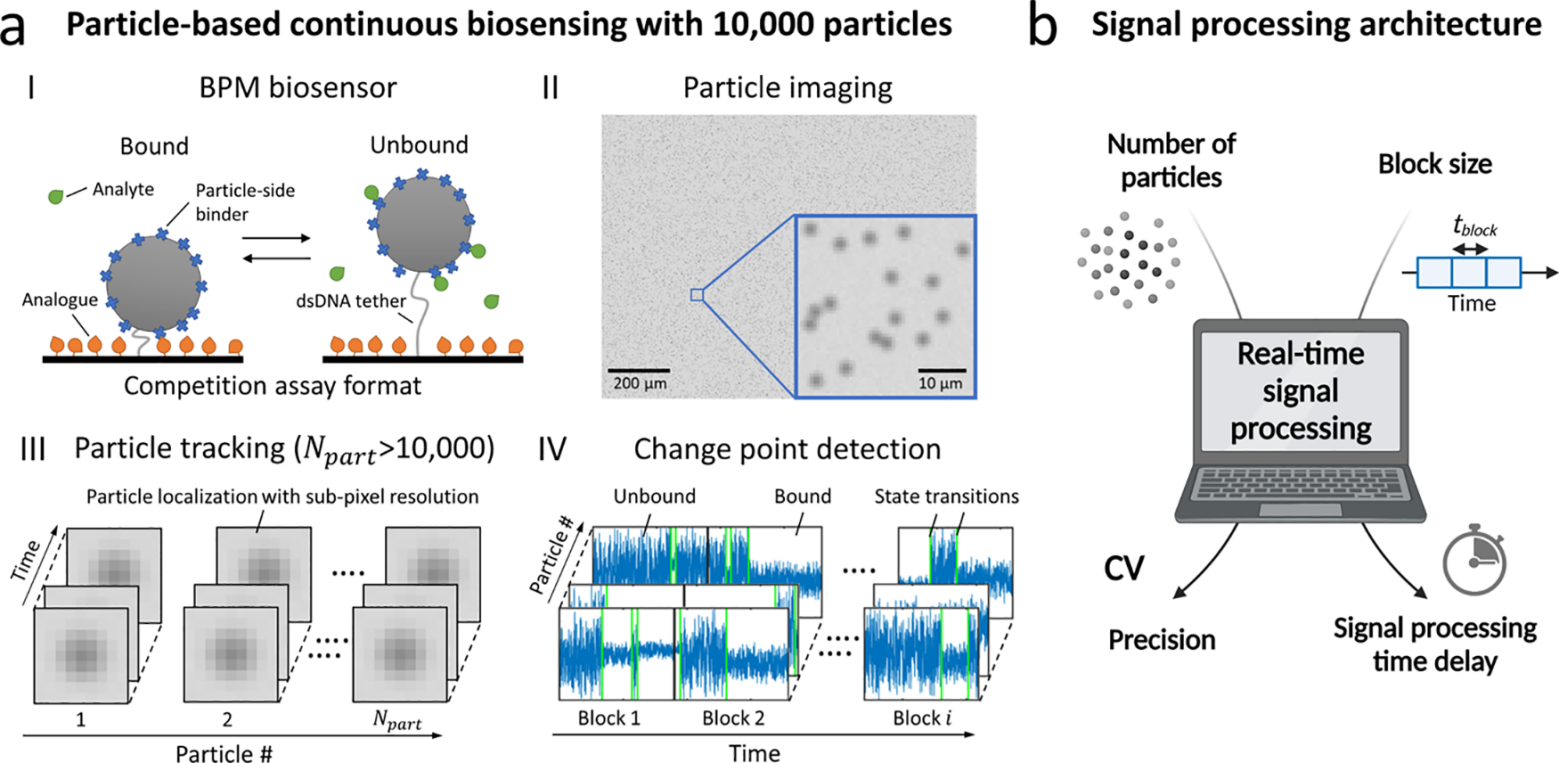
A signal processing architecture is needed to enable real-time continuous biosensing based on high-throughput single-molecule sensors, here exemplified with BPM. (a) BPM sensing with 10,000 particles. (I) Sketch of a BPM biosensor with competition assay. Biofunctionalized particles interact reversibly with a biofunctionalized substrate. The rate of switching between bound and unbound states depends on the analyte concentration in solution. (II) Particles are imaged with video microscopy. The image shows more than 10,000 particles. In this study, particles are used with a diameter of 1 μm. (III) Particle tracking is performed by computing the positions of individual particles in consecutive video frames. (IV) Position time traces of individual particles show transitions between states with different mobilities. A change point detection (CPD) algorithm detects state transitions in the obtained *x* and *y* time traces. The state transitions are detected within consecutive measurement blocks with a predefined block size *t*_block_. (b) The signal processing architecture enables continuous real-time measurements with control of the trade-off between measurement precision and time delay. Input parameters are the number of particles *N*_part_ and the block size *t*_block_. Output parameters are the precision and the signal processing time delay Δ*t*_SP_.

Figure 1 illustrates the real-time signal processing challenge for high-throughput single-molecule sensors, exemplified with BPM. Concentrations of analyte molecules are measured in a BPM biosensor by analyzing the motion of particles with a typical diameter of 1 μm. In a BPM sensing experiment, an output parameter that relates to the analyte concentration is the switching activity, which is the average number of state transitions per particle per unit of time. The activity is determined in defined time intervals or measurement time blocks with a block size *t*_block_ and is given by
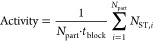
1

Here, *N*_part_ is the number of tracked particles, and *N*_ST,*i*_ is the number of state transitions of particle *i* within the measurement block. The motion of particles is tracked by video microscopy. For each particle, *x* and *y* time traces are constructed from the localizations in consecutive video frames. The number of state transitions in each measurement block can be determined by applying a change point detection algorithm to the time traces. Figure 1b explains the need for a signal processing architecture for analyzing the BPM data. For continuous biosensing applications, generating a real-time response with short time delays is essential. The total time delay of the real-time biosensor has contributions from physical and biochemical processes in the biosensor, such as transport of molecules in the fluid and the binding and unbinding of molecules,^31^ as well as from the signal processing that is required to translate recorded particle motion traces into a concentration–time profile.

In this paper, we focus on the topic of signal processing, i.e., the signal processing time delay. In order to achieve a short signal processing time delay, a small block size is needed. However, a small block size decreases the measurement statistics and the precision because the number of observed state transitions per particle is reduced. The statistics can be increased by increasing the number of analyzed particles, which can also be beneficial for purposes such as multiplexing^22^ and measurements over long time spans.^24,32^ However, increasing the number of analyzed particles leads to an increased computational cost. This requires a computational system with a larger size and power consumption, which complicate the miniaturization and wireless applications of a biosensor. Thus, it is important to design a real-time signal processing architecture that is suited for small block sizes and large numbers of particles, that operates with high computational efficiency, and that gives control of the trade-off between time delay and measurement precision.

Recording particle motion in a large field of view (FOV) at a typical frame rate of 30 Hz involves image data streams of several gigabytes per minute. Using a previous data analysis approach,^21^ the total computation time required for particle tracking and change point detection was typically several hours for a few minutes of video data. Such an approach is suited for post-processing after the completion of an assay experiment, but not for continuous monitoring applications where a short signal processing time delay and significant data reduction are desired. Thus, a real-time signal processing architecture is needed that includes image acquisition and computationally efficient methods for particle tracking and detection of state transitions.

One of the computationally demanding parts of BPM signal processing is the tracking of many particles, where each particle represents a single-molecule probe. Particle tracking methods have been described in the literature,^33,34^ but these were not designed for efficient computation. For example, Cnossen et al. demonstrated a method for real-time tracking of hundreds of particles with nanometer accuracy at a frame rate of 60 Hz.^33^ Quadrant interpolation was applied for 3D localization, which relies on large magnifications and large regions of interest (ROI) around each particle of typically 100 × 100 pixels. These large ROI sizes limit the analysis to only several hundreds of particles in a FOV. In addition, the method requires the use of a high-performance graphics processing unit (GPU), which limits the flexibility and is demanding on processor size and power consumption.

In contrast, we aim to develop a signal processing architecture that can run on a central processing unit (CPU) of a standard laptop computer rather than on a high-performance GPU, because this will provide high flexibility and will make the signal processing architecture compatible with future sensor implementations in wireless and wearable devices. In the next sections, we will discuss the developed signal processing architecture, the validations, and the real-time continuous biosensing experiments.

### Signal Processing Architecture

**Figure 2:**
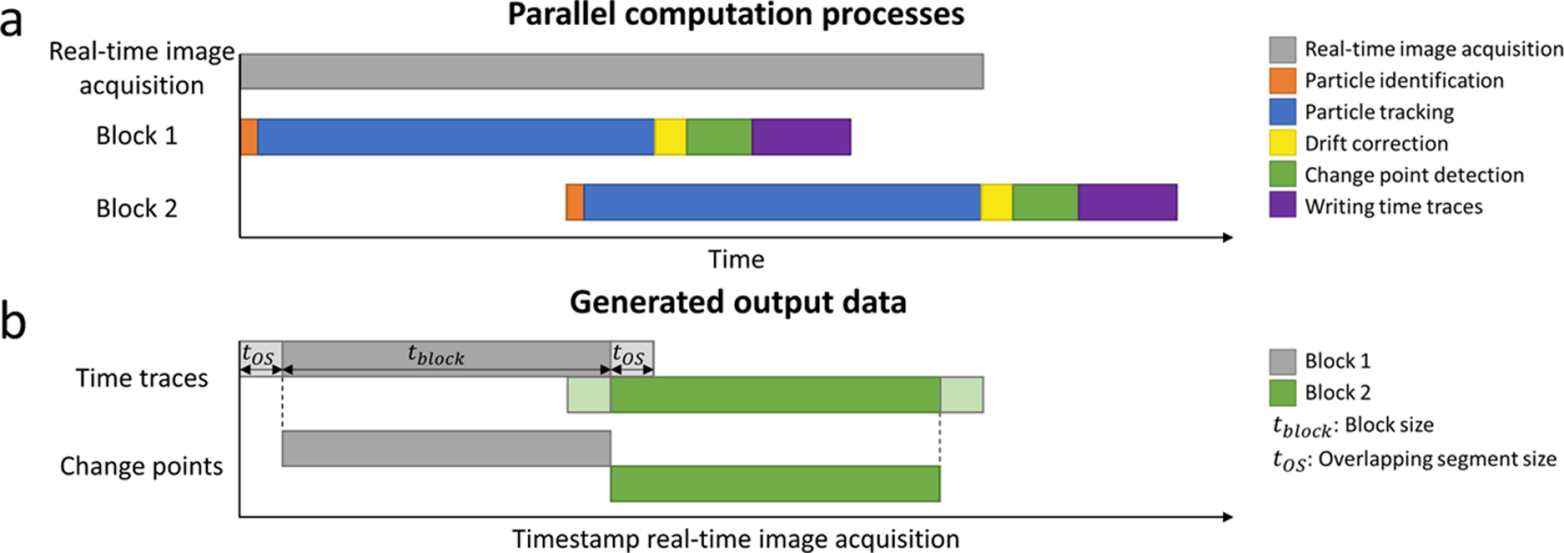
Signal processing architecture for real-time continuous biosensing: time structure of parallel computation processes and generation of output data. (a) Real-time execution of parallel computation threads. One thread is created for real-time image acquisition. For each measurement block, a new thread is started as soon as the first image of the measurement block is available. In each block, a series of data analysis steps is performed, as indicated by the colors. (b) Generated output data consist of *x* and *y* time traces and change points. Time traces of consecutive blocks with block size *t*_block_ have overlapping segments with size *t*_OS_.

Figure 2a shows the basic structure of the signal processing architecture for high-throughput analysis of single-molecule BPM sensors. The application of the signal processing architecture to other single-molecule sensing techniques will be elaborated in the Results section. Computation processes are performed in parallel to enable long continuous measurements in real time and to be able to exploit the full capacity of the CPU. Computation tasks are divided into threads that can be executed at the same time, which is referred to as multithreading. Figure 2a shows that one computation process, or thread, is created for real-time image acquisition, i.e., capturing frames from the camera in real time. At the same time, frames that have been captured can be processed by other threads. We developed a programming structure with multiple measurement blocks that enables continuous measurements, in principle, over an endless time span. In each measurement block, a sequence of data analysis steps is performed, which will be described in this section. Measurement blocks have a predefined block size *t*_block_, and each block has overlapping segments *t*_OS_ on both sides. The overlapping segments are required to provide reliable detection of state transitions near the boundaries of a block, which will be elaborated further in the Results section. Figure 2b shows the generated output data, showing overlap between the generated time traces of consecutive blocks. The block structure has several advantages. It reduces memory usage since the memory occupied by a block is cleared after the block is finished. It also provides a structured way for generating real-time output data at defined time intervals. Furthermore, the block structure allows the acceptance of new particles that might move into the FOV due to drift or diffusion during the measurement. The new particles can be tracked in the next measurement block since particles are identified at the start of each measurement block.

### Real-Time Software Framework

The signal processing architecture is implemented in a CPU-based software framework. The software framework has been developed in Microsoft Visual Studio 2015 in the C++ programming language and has an object-oriented programming structure to achieve high computational efficiency and allow detailed control of multithreading and memory management.

### Real-Time Image Acquisition

Real-time image acquisition is performed by capturing individual frames from a video of moving particles. The software framework also allows reading in frames from a storage device, making it also suited for post-processing or for performing validations.

### Particle Identification

In the first frame of a measurement block, particles are identified by defining a region of interest (ROI) around each individual particle, which acts as a single sensor probe. The particle identification is required to exclude particles that cannot be localized accurately, e.g., particles that are too close to neighboring particles. The particle identification is designed to be robust for images with a wide range of areal particle densities and involves a sequence of steps that include Gaussian smoothing, local thresholding, filtering to exclude particles with close neighbors, and filtering to exclude ROIs with deviating intensities. Supporting Information 1 describes the details of the particle identification methods.

### Particle Tracking

Particle tracking includes localizing the *x* and *y* positions of the particles as a function of time. In each frame, particle positions are determined with sub-pixel resolution by applying a localization algorithm to the ROI around each particle. A 2D localization method is used with a small ROI around each particle to enable the tracking of a large number of particles. Localizing particles with small ROI sizes is similar to localizing fluorophores in single-molecule localization microscopy, where several algorithms are applied, such as Gaussian fitting,^35,36^ centroid fitting,^37^ and radial symmetry.^38^ Here we use phasor-based localization, as this is computationally extremely efficient and provides accurate localization for ROI sizes that are as small as 5 × 5 pixels.^39^ An important assumption in the particle tracking is that particle movement is limited between two consecutive frames, as this allows to use the ROIs in frame *i* as input for frame *i* + 1. In the particle localization of each single frame, it is crucial that the position of the ROI is updated if the localized particle position is far from the center of the ROI. This prevents particles to move outside the ROI in a series of consecutive frames. An advantage of this implementation is that particle identification only needs to be performed at the start of a measurement block, which is beneficial for computational efficiency. Additionally, there is no need for linking algorithms that are typically used to construct individual particle time traces by correlating localizations from multiple frames.^40−42^ There are two scenarios in which the tracking of identified particles is discarded. This occurs if the corresponding ROI requires more than five updates in a single frame, preventing a deadlock by infinitely updating ROIs in a single frame. The tracking is also discarded if a part of the ROI moves outside the FOV, which might happen due to sample drift or particle movement.

### Drift Correction

Particle *x* and *y* time traces are corrected for drift by subtracting the change in average position of a selection of particles. The selected particles are preferably immobilized particles or particles with small movements, since their motion is a more precise indication for the drift in the sample. Supporting Information 2 explains how the drift correction method selects a subset of particles to calculate the drift in the sample.

### Change Point Detection

State transitions are detected in the drift-corrected time traces by applying the maximum-likelihood multiple-windows change point detection (MM-CPD) algorithm.^43^ The algorithm calculates the probability of a change in distribution by comparing the distributions of neighboring windows of data points in a time trace. The approach combines multiple window sizes to achieve reliable detection of state transitions in time traces with multiple heterogeneities, such as distributions, lifetimes, and time-correlation properties. The MM-CPD algorithm has three algorithm parameters that control the CPD performance: the minimum window size *w*_min_, the number of windows *N*, and the threshold. The data in this research are analyzed with *w*_min_ = 20, *N* = 5, and a threshold of 25, unless stated otherwise. One key advantage of the MM-CPD algorithm is that it is computationally very efficient and is therefore suitable for real-time high-throughput measurements.

### Writing Time Traces

The final step in a measurement block is to store the relevant output data. The output that is stored after each measurement block consists of text files with the *x* and *y* time traces, the calculated drift, and the detected state transitions of each particle. Storing the CPD data is done prior to storing the time traces, since this data is directly needed as a real-time output of the biosensor.

### Simulations and Quantitative Evaluation

The implemented data analysis methods were validated by analyzing simulated data. This approach allows quantitative evaluations since the output of the signal processing can be compared with the ground truth simulated data. The simulation includes typical physical parameters corresponding to the BPM system, including the diffusion coefficient, unbound and bound state position distributions and lifetimes, and radial intensity profiles. Sequences of images are constructed with in each frame individual particles on the positions corresponding to the simulated particle *x* and *y* time traces. Each particle is represented by a radial intensity profile extracted from experimental images obtained with video microscopy, which results in a close match between simulated and experimental images. Supporting Information 3 explains these simulations in more detail.

The evaluation parameter for particle identification and localization is the root-mean-square error (RMSE) or the localization error of particles
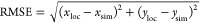
2where *x*_loc_ and *y*_loc_ represent the position coordinates localized by the software framework of a single particle, and *x*_sim_ and *y*_sim_ are the simulated positions. In addition to the localization error, the number of identified particles is an evaluation parameter for the particle identification since it is desired to identify a large number of particles that can be localized with high accuracy.

### Experiments for Real-Time Continuous Biosensing of Cortisol Using BPM

#### Materials

The oligonucleotides employed in this research were obtained from Integrated DNA Technologies (IDT). Reagents were acquired from Sigma, and cartridges were purchased from Ibidi. Cortisol-DNA conjugates and antibody biotinylation were prepared as described by van Smeden et al*.*^26^

#### Sensing Surface Functionalization

Ibidi cartridges (μ-Slide III 3-in-1, Ibidi) were cleaned by 10 min sonication in Milli-Q water. Subsequently, the cartridges were dried with a nitrogen stream and exposed for 30 min to UV ozone treatment (Digital UV Ozone System, Novascan), followed by cartridge sealing (Sealing tape, Thermo Scientific). Afterward, the fluidic channel was functionalized with PLL-g-PEG polymers as described by Lin et al.^24^ and van Smeden et al*.*^26^ Then, 50 μL 0.5 nM of 221 bp dsDNA tether, and 50 μL 2 μM ssDNA-DBCO were added to the fluidic channel to finish the surface functionalization (molecular details described by van Smeden et al.^26^).

#### Particle Functionalization

Streptavidin-coated magnetic particles with 1 μm diameter (Dynabeads MyOne Streptavidin C1, Thermo Scientific) were functionalized as described by van Smeden et al.^26^ but with some changes to the protocol. Particles were incubated with 250 nM of biotinylated anti-cortisol antibody and 1.5 μL of 10 μM polyT. The functionalized particles were washed twice with 500 μL of 0.05% Tween-20 in PBS buffer and followed by a sonication step in a sonication bath for 30 s.

#### Sensor Assembly

200 μL of functionalized particles were flushed (Harvard pump 11 Elite, 100 μL/min withdrawal speed) through the Ibidi flow cell (Ibidi). The particles were incubated for 15 min by flipping the flow cell to allow the particle binding via dsDNA tethers. 200 μL of 100 μM of 1 kDa mPEG-biotin (PG1-BN-1k, Nanocs) was added to the system and incubated for 10 min in order to block the remaining free streptavidin molecules. The motion of the tethered particles was recorded during the blocking step to establish the signal background of the system. Unless stated otherwise, 200 μL of 700 pM cortisol-DNA (analogue) was added to the flow cell and incubated for 10 min to allow the system activation. The excess of analogue was removed by flushing with 0.5 M NaCl in PBS buffer and followed by the addition of different cortisol solutions prepared in 0.5 M NaCl in PBS buffer.

#### Measurement Setup and Flow Protocol

Particles were imaged with brightfield microcopy on a Leica DM6000 B microscope with a total magnification of 5.5 (Objective: Leica N plan EPI 10*x*/0.25 BD, C-mount: 0.55*x*). A high-speed CMOS camera was used (FLIR GS3-U3-32S4M-C) with a FOV of 2048 × 1536 pixels (1.28 × 0.96 mm^2^). Solutions were flown into the cartridge at a flow rate of 100 or 10 μL/min for BPM measurements using a syringe pump (Harvard Apparatus Pump 11 Elite) connected to the outlet of the flow cell.

## Results and Discussion

### Quantitative Evaluation of the Signal Processing

**Figure 3:**
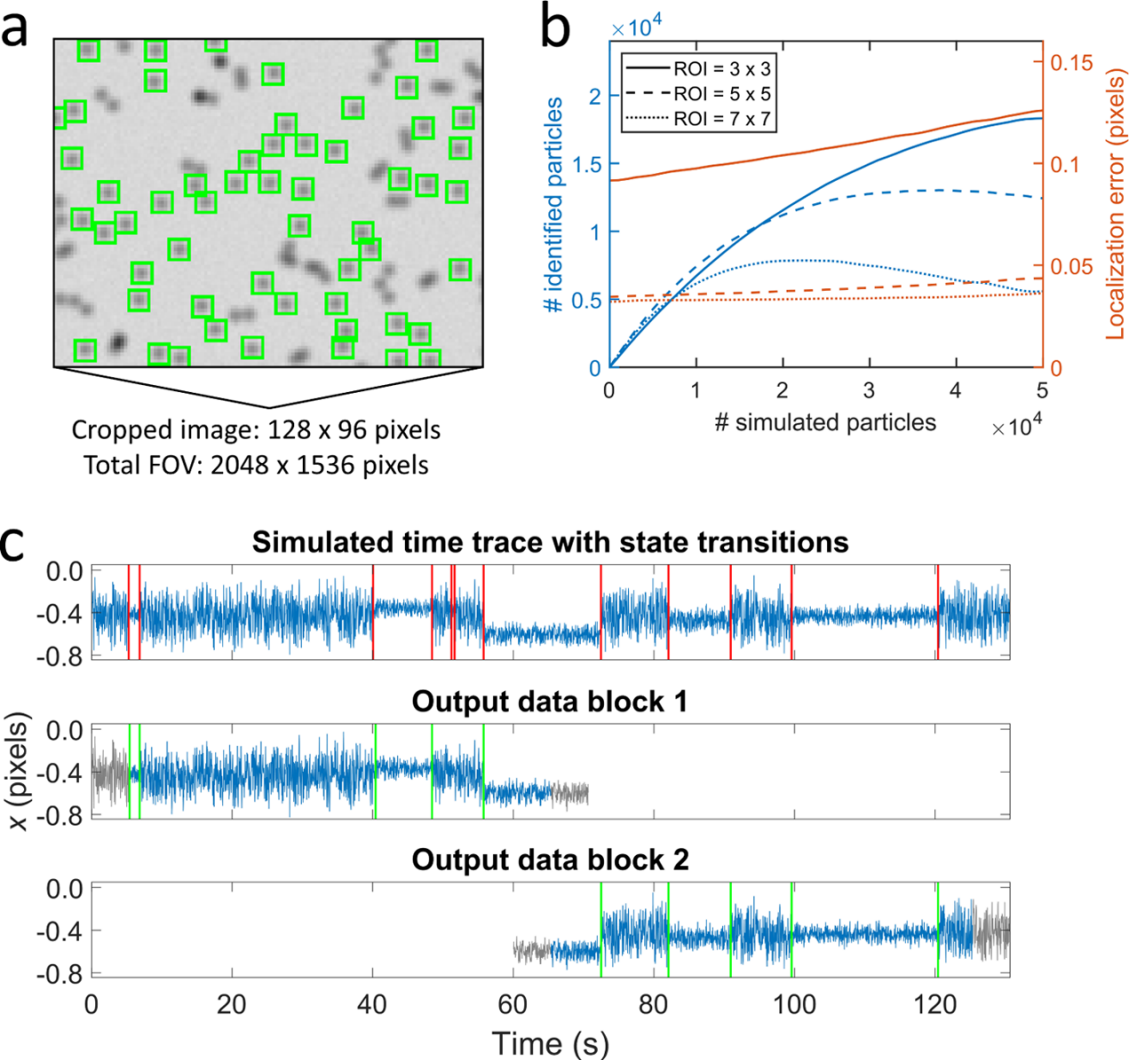
Evaluation and validation of the signal processing with simulated data. (a) Cropped image of a simulated field of view (FOV) with a high areal density of particles. The green squares indicate the identified particles. (b) The number of identified particles (blue, left axis) and the average localization error (red, right axis) as a function of the number of simulated particles in a FOV of 2048 × 1536 pixels. The three line styles correspond to the different ROI sizes as shown in the legend. (c) The top panel shows a simulated time trace of the *x* position of a BPM particle. The red vertical lines indicate the simulated state transitions. The middle and bottom panels show the generated output data by the software framework for block 1 and block 2, respectively. The output data consist of time traces with detected state transitions (green vertical lines). The gray parts in the time trace indicate the overlapping segments between consecutive blocks. See Supporting Information 4 and 5 for details and quantitative evaluations of particle tracking, drift correction, and change point detection.

Figure 3a shows a cropped image of a simulated FOV with a high areal density of particles. The complete FOV is 256 times larger and corresponds to a typical experimental image in BPM, as is shown in Figure 1a–II. The green squares indicate the identified particles for localization with an ROI size of 5 × 5 pixels. The data show that particles with close neighbors are not identified. Figure 3b shows the average localization error (eq 2) and the number of identified particles as a function of the number of simulated particles for different ROI sizes. A significantly larger localization error is observed with an ROI size of 3 × 3 pixels compared to the larger ROI sizes. This increased localization error is most likely due to a loss of information since a part of the intensity profile corresponding to the particle is outside the ROI. Furthermore, it is observed that the average localization error only slightly increases as a function of the number of simulated particles, which indicates that the particle identification methods are robust for high areal particle densities. The number of identified particles in a FOV is clearly dependent on the ROI size. Smaller ROI sizes result in the identification of more particles since the probability that the intensity profile of a neighboring particle is inside the ROI of a particle decreases. For all ROI sizes, the number of identified particles increases as a function of the number of simulated particles until a maximum number of identified particles is reached. For simulated areal particle densities above this maximum, the number of identified particles decreases due to a larger fraction of particles with a close neighbor. For the used conditions, an ROI size of 5 × 5 is considered as the best choice to achieve both a large number of identified particles and a small localization error. For example, a simulated FOV with 20,000 particles results in ∼12,000 identified particles with an average localization error of ∼25 nm (∼0.04 pixels).

Generation of *x* and *y* time traces includes particle tracking, i.e., localization of particles as a function of time, and drift correction. Particle tracking can be evaluated by analyzing sequences of simulated images and comparing the simulated time traces to the detected time traces, which is shown in Figure 3c. In this example, no drift is present in the simulated time trace. Supporting Information 4 includes further validations of the particle tracking and drift correction, showing, for example, that the drift correction error with ∼10,000 tethered particles was approximately one order of magnitude smaller compared to the particle tracking error. This indicates that an accurate drift correction can be achieved with only tethered particles and that additional fiducial markers are not needed.

Figure 3c also shows the simulated and detected state transitions in the time traces. The blue-colored parts of the time trace indicate the segments where state transitions are detected, the green vertical lines represent the detected state transitions, and the gray parts of the time trace indicate the overlapping segments between consecutive blocks (see also Figure 2). The overlap is required because state transitions near the boundary of a time trace are less likely to be detected. Supporting Information 5 shows that the required overlapping segments should be two times larger than the largest window size that is applied in the MM-CPD algorithm. The largest window size is defined by the MM-CPD settings, e.g., *w*_min_ = 20 and *N* = 5 gives a largest window size of 80 data points,^43^ so overlapping segments of 160 data points are required. By implementing these overlapping segments in the signal processing architecture, the detected change points are independent of the block size that is chosen.

### Real-Time Computational Performance

The real-time computational performance was assessed by running the software framework and measuring the CPU utilization with the Intel VTune Profiler. The computational performance measurements were performed on a laptop with Microsoft Windows 10 Enterprise and an Intel Core i7-8750H processor (6 cores, 12 logical processors, 2.20 GHz, 16 GB RAM). The profiling measurements were performed in real time during a BPM experiment where ∼10,800 particles were analyzed. The particles were tracked and analyzed for 3 min at 30 Hz with a block size of 1 min.

**Figure 4:**
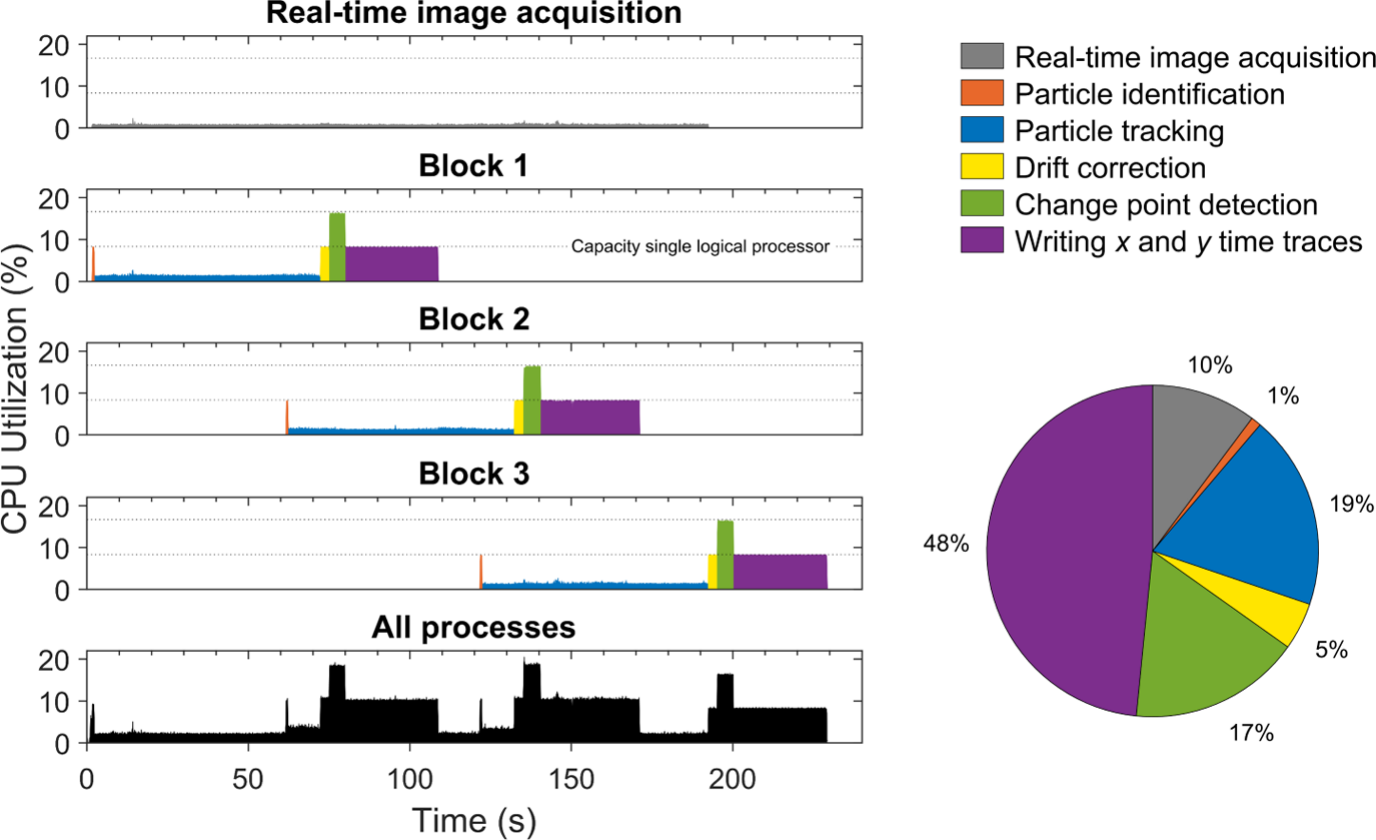
Real-time computation characteristics recorded during a BPM experiment with ∼10,800 analyzed particles that were imaged at a 30 Hz frame rate. The top four panels indicate the CPU utilization as a function of time for different parallel computation processes. The dotted lines indicate the maximum capacity of one and two logical processors. Data analysis steps in each measurement block are indicated by color. The bottom panel shows the sum of the CPU utilization of all processes. The pie chart shows the percentage of CPU utilization that is spent on each data analysis step.

Figure 4 shows the CPU utilization for the different parallel computation processes that were discussed in the Methods section. Real-time image acquisition was performed at a rate of 30 Hz and required only ∼0.8% CPU utilization. The different serial processes in a measurement block are indicated by color and can also be recognized by their computational performance. During particle identification, drift correction and writing the *x* and *y* time traces the CPU utilizations are close to 8.3%, which is the maximum capacity of a single logical processor (100% is the total capacity of the laptop, having 12 logical processors). During particle tracking, the CPU utilization of the measurement block is less than the maximum capacity of a single logical processor, which indicates that a significant fraction of the time the thread is waiting for new images to arrive. Thus, the localization of all particles in a single image is already completed before the next image is available. During change point detection (CPD), the CPU utilization of the measurement block is approximately two times the maximum capacity of a single logical processor. This is because a multithreading approach is applied with one extra worker thread. Applying a multithreading approach leads to a faster generation of the CPD results and thus the output signal. The pie chart in Figure 4 shows that most CPU time is spent on writing the *x* and *y* time traces. The text files containing the time traces for these 3 measurement blocks are ∼1.0 GB in size. Storing this data is useful for post-processing in research applications, e.g., for determining state lifetimes. In real-time biosensing applications, this step could be avoided, leading to a significant decrease in CPU utilization. The bottom panel shows the total CPU utilization, which is obtained by taking the sum of the CPU utilizations of all parallel computation processes. The total CPU utilization is on average only ∼6.5% in this measurement during ∼230 s. To get a representative value for long continuous measurements, we need to correct for the fact that the total running time is longer than 3 min, applying this correction gives an average effective CPU utilization of ∼8.3%. This value is approximately equal to the capacity of a single logical processor, indicating that real-time measurements with more than 10,000 particles at 30 Hz can easily be performed on a laptop with several logical processors.

We can express the total signal processing time delay Δ*t*_SP_ as a sum of different contributions
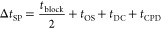
3

Here, Δ*t*_SP_ is equal to the time difference between the time at the middle of a measurement block and the time at which the CPD results of the block are available. Δ*t*_SP_ depends on the block size *t*_block_, the overlapping segments *t*_OS_, and on the computational time of the drift correction *t*_DC_ and change point detection *t*_CPD_. The computational time of particle tracking does not contribute to the signal processing time delay since the particle tracking is executed in real time. The activity (eq 1) is available directly after the CPD is performed; storing the CPD results takes a negligible amount of time and is done prior to storing the *x* and *y* time traces. Choosing a smaller block size generally leads to a shorter Δ*t*_SP_. However, choosing a block size smaller than *t*_OS_ leads to a strong increase in CPU utilization due to the overlapping segments between consecutive blocks (see Supporting Information 5). *t*_DC_ and *t*_CPD_ could be decreased by improving the computational efficiency of the drift correction and CPD methods. Alternatively, multithreading approaches can be implemented, as was already done for CPD (see Figure 4).

### Real-Time Cortisol Biosensing

**Figure 5:**
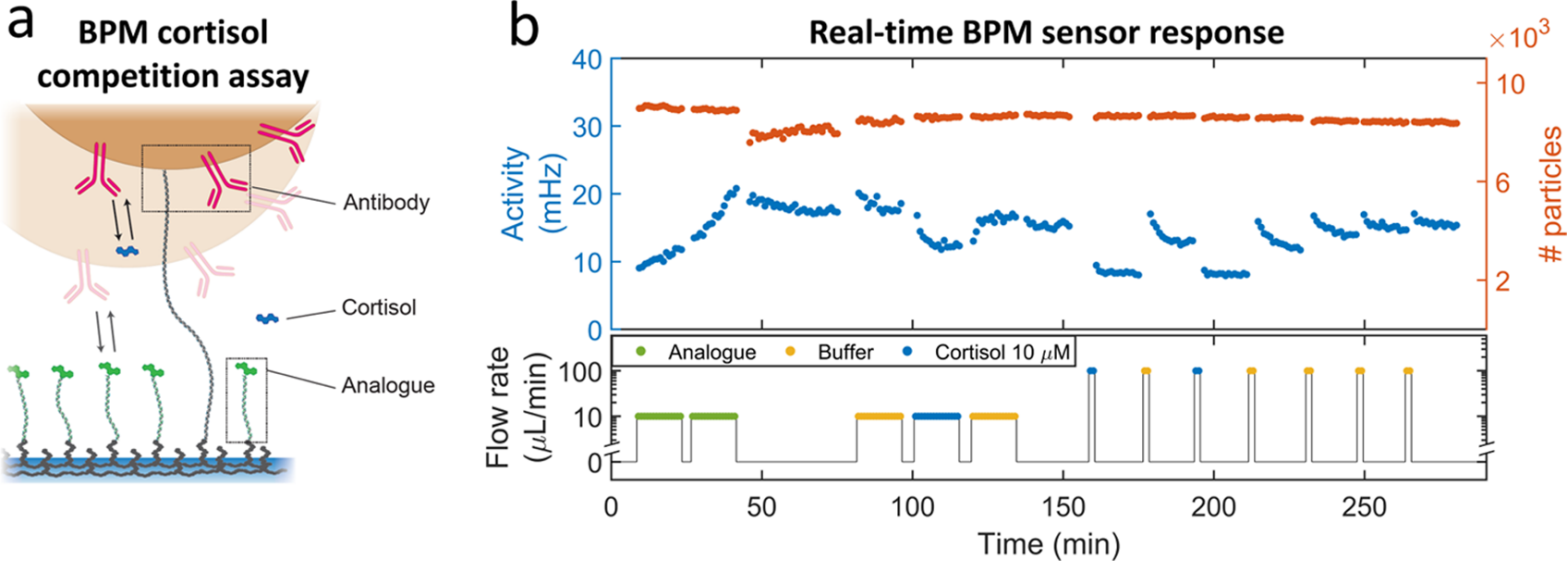
Real-time measurements with a cortisol BPM sensor. (a) Design of the cortisol competition-assay biosensor that was used to test the real-time data analysis. Cortisol-analogue molecules are coupled to the substrate and anti-cortisol antibodies to the particle. Reprinted with permission from van Smeden et al*.*^26^ Copyright 2022 ACS Sensors. (b) Real-time measurements. The bottom panel indicates the flow protocol, showing how the sensor was exposed to different solutions as a function of time: analogue (green), buffer (yellow), and 10 μM cortisol (blue). The top panel shows the measured activity (blue, left axis) and the number of particles (red, right axis) as a function of time. Each dot represents the activity value within a measurement block of 1 min (see Supporting Information 6 for details).

In order to demonstrate the real-time data analysis experimentally, we performed real-time cortisol measurements in a BPM sensor with a competition assay format (see Figure 5a). A permanent dsDNA tether confines the motion of a particle to the vicinity of the substrate. Cortisol analogues are coupled to the substrate, and anti-cortisol antibodies are coupled to the particles. During sensor operation, in the absence of cortisol in solution, reversible bonds are formed between analogue molecules on the substrate and antibodies on the particles. In the presence of cortisol in solution, cortisol binds to the antibodies on the particle and thereby blocks the interaction between the analogue molecules on the substrate and the antibodies on the particle. Thus, a higher concentration of cortisol results in a decreased probability of bond formation between the particle and the substrate, and therefore the activity decreases, i.e., the number of state transitions per unit of time decreases.

Figure 5b shows an experiment where the response of a cortisol sensor was followed in real time. The sensor was first provided with analogue molecules (green) in order to activate the sensor; thereafter, buffer solutions (yellow) and solutions with 10 μM cortisol (blue) were flown into the sensor cartridge. The bottom panel shows the applied flow profile. In each step, measurements were performed consisting of 15 blocks of 1 min. The activity values plotted in Figure 5b were filtered to suppress measurement artifacts due to flow instabilities, see Supporting Information 6.

Before the addition of analogue, the measured activity is approximately 10 mHz. This background activity is caused by non-specific interactions and false positive events in the change point detection.^43^ During the addition of analogue solution at a flow rate of 10 μL/min, the switching activity increases, indicating that analogue molecules bind to the substrate, causing reversible bonds to be formed between analogue molecules on the substrate and antibodies on the particles. In the next two phases (absence of flow between 40 and 80 min; flow of buffer between 80 and 95 min), the activity is largely constant, with a small and slow downward relaxation. The origin of the small relaxation is not yet clear; this will be addressed in future work. When 10 μM cortisol is supplied, the activity drops rapidly and reaches an equilibrium level, indicating that cortisol binds to the antibodies on the particle. The addition of buffer solution causes the activity to rapidly increase, caused by the dissociation of cortisol from the antibodies.

The real-time continuous biosensor allows studies of sensor response to fluid pulses. From ∼160 min, a series of pulses was applied with 2 min duration each and a flow speed of 100 μL/min, containing either buffer or 10 μM cortisol. After stopping the flow, we noticed in the fluidic system a residual flow with a duration of typically 1–10 s, caused by relaxations of the pump and tubing. For that reason, we inserted a waiting time of 1 min between the termination of sample injection and the start of the activity measurements. In the measured activity, we observe different relaxation behaviors after injections with buffer and 10 μM cortisol. The sensor signal is rapidly in equilibrium after exposure to 10 μM cortisol. After exposure to buffer, the measured activity signal first increases and then shows a relaxation toward lower activities. We call this a reversed relaxation. The reversed relaxation indicates fast dissociation of cortisol during the 2 min fluid pulses, followed by slow association of cortisol during the 15 min measurements without flow. The data show that the degree of reversed relaxation becomes less and less after multiple injections with buffer. We attribute the post-buffer signal relaxations to the lateral diffusion of cortisol into the FOV from edges and corners of the flow cell. Repeated inflow of buffer is required to remove cortisol from the complete sensor cartridge and achieve a stable BPM signal. This indicates that a larger volume of fluid is needed to achieve a sensor with a uniform zero analyte concentration compared to a high analyte concentration.

**Figure 6:**
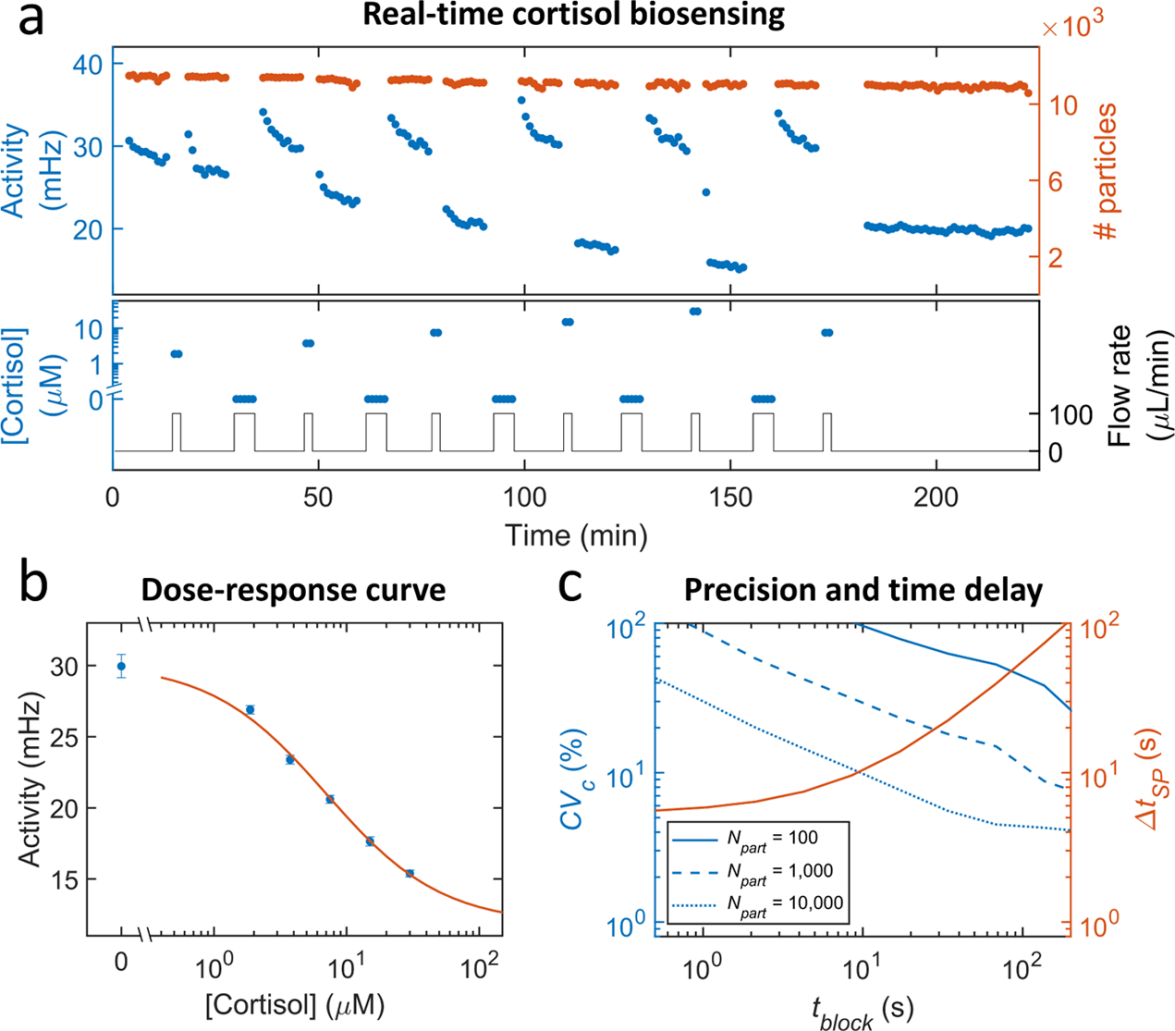
Analytical performance of a real-time cortisol BPM biosensor: concentration measurement precision and signal processing time delay. (a) The bottom panel shows the series of different cortisol concentrations (blue, left axis) alternated with a blank buffer solution that were supplied to a BPM biosensor. The flow rate (black, right axis) was 100 μL/min for a duration of 2 and 5 min for cortisol solution and buffer solution, respectively. Prior to the measurements, the sensor was activated by flowing analogue molecules for 2 min at 100 μL/min, causing a higher switching activity compared to the experiment of Fig. 5. The top panel shows the measured activity (blue, left axis) and the number of particles (red, right axis) as a function of time. Each dot represents the obtained value in a measurement block of 1 min. (b) Dose–response curve established using the data of panel a. The average was determined from the last five data points at each concentration. The error bars indicate the standard deviation of the data points. The zero concentration datapoint and its error were determined from multiple zero-analyte measurements in panel a. Datapoints were fitted with a sigmoidal curve (see Supporting Information 7), resulting in an EC50 value of 7 μM. (c) Trade-offs between precision and time delay of the real-time biosensor. Left axis: coefficient-of-variation of concentration determination CV_C_ (see Supporting Information 7 for details), plotted as a function of the block size for different numbers of particles. CV_C_ was determined from the signal variation of the 40 min measurement at 7.5 μM shown in panel a and the slope of the dose–response curve shown in panel b. Right axis: signal processing time delay Δ*t*_*SP*_ as a function of the block size. Here, *t*_*OS*_ was included and times required for drift correction and change point detection were neglected, see eq 3.

The trade-off between precision and time delay is studied in Figure 6. Figure 6a shows the real-time activity measured for a series of different cortisol concentrations alternating with a blank buffer solution. The flow was 100 μL/min for a duration of 2 and 5 min for cortisol solution and buffer solution, respectively, because a zero analyte concentration requires a larger volume of fluid to achieve a uniform analyte concentration in the sensor (cf. Figure 5). Thereafter, measurements with 10 blocks of 1 min were performed without flow. From 180 min onward, a long measurement was performed consisting of 40 blocks of 1 min. As in Figure 5, a relaxation behavior is seen in the signal, most notably after the supply of fluid with low cortisol concentrations and less after high cortisol concentrations, caused mainly by diffusive transport effects;^31,32^ these effects will be further studied in future research. Figure 6b shows the dose–response curve, which was determined by averaging the activity values in the last 5 min at each concentration. The datapoints follow a sigmoidal curve for a competitive assay (more details are given in Supporting Information 7).

A real-time continuous biosensor has a trade-off between measurement precision and time delay because a sensor with shorter time delay can be achieved by choosing a smaller block size, resulting in a lower precision due to lower measurement statistics. The blue lines in Figure 6c show the coefficient of variation of concentration determination (CV_C_) as a function of the block size used in the computation, plotted for different numbers of particles. The CV_C_ was determined from the fluctuations of the activity signal measured in panel a and the slope of the dose response curve in panel b at a cortisol concentration of 7.5 μM; see Supporting Information 7 for more details.

The graph shows essentially straight lines with slopes close to −0.5, indicating an inverse square-root behavior; this is in agreement with a system in equilibrium that obeys Poisson statistics. Furthermore, the CV_C_ scales with *N*_part_^–1/2^, which is also in agreement with Poisson statistics. The blue curves show deviations from straight lines at large values of *t*_block_; the variability can be attributed to the small number of datapoints that are available for estimating CV_C_ at large block sizes. The red curve shows that the signal processing time delay is independent of *t*_block_ for small block sizes and approaches *t*_block_/2 for large block sizes.

A continuous biosensor is a real-time biosensor when it reports sensing signals with a time delay that is short with respect to the typical dynamics of the biomarker concentration in the system that is being monitored. The acceptable time delay depends on the application. In the case of cortisol, the biomarker is a steroid hormone that is produced by adrenal glands and affects tissues and organs all over the body. Cortisol levels can increase on timescales of minutes to tens of minutes, and decrease on longer timescales. The data in Figure 6c show that suitable time delays are achievable. With the help of data as in Figure 6c, sensor users can choose signal processing settings (block size, number of particles) in order to achieve desired functional performance parameters of the single-molecule biosensor (time delay, precision). For example, a sensor with a CV_C_ of 10% and a signal processing time delay of about 10 s can be achieved with a sensor consisting of 10,000 particles and a block size of 10 s.

### Application to Other Single-Molecule Sensors

Single-molecule biosensors give signals with characteristic states and transitions which originate from single-molecule interactions, e.g., on/off states, discrete signal levels, number of transitions, etc. Single-molecule sensors combine signals from multiple single-molecule probes in order to collect sufficient statistics in a limited amount of time. Table 1 lists a few single-molecule sensing principles that could become suited for real-time continuous biosensing: based on detecting fluorescently labeled molecules, on measuring spectral shifts of plasmonic particles, and on measuring electrical conductivity fluctuations of nanopores. The methods yield parallel signals originating from individual single-molecule probes, where the signals are time traces with discrete states and the concentration of analyte molecules in solution changes the time characteristics of the signals, e.g., distributions of state lifetimes. The last column indicates how the sensor signals have been analyzed in the literature. To turn these methods into real-time continuous sensors, the time-dependent signals need to be available in high throughput, so that sufficient single-molecule statistics can be collected in a limited amount of time. The principle is similar to traditional biosensors with a single readout parameter, such as a current or a light intensity, where the signal is averaged over a certain time window in order to achieve a sufficiently large signal to noise ratio. In single-molecule sensors, signals are collected from a large number of fluorophores, plasmonic particles, nanopores, or BPM particles, and these signals need to be efficiently processed in a limited amount of time. Furthermore, the signal processing methodology must be suited for measurements over an endless timespan, with control of the trade-off between signal processing time delay and analytical precision.

**Table 1:** Examples of Single-Molecule Sensing Methods to Which the Developed Signal Processing Architecture Could be Applied for Achieving Real-Time Continuous Biosensing

single-molecule sensor	single-molecule probe	physical signal	analysis of signal time traces
fluorescence sensor^15^	fluorescently labeled detection molecule	light intensity (fluorescence)	hidden Markov modeling to extract the number of binding events and dwell times
plasmonic sensor^44^	gold nanoparticle	light intensity (scattering)	step finding to extract waiting times between binding events
nanopore sensor^45^	nanopore	electrical conductivity	extract event frequency and analyze dwell times to identify current signatures
BPM sensor (demonstrated in this paper)	tethered particle	position	change point detection to extract rate of state transitions

In this paper, we have described a signal processing architecture and applied it to BPM, turning the single-molecule method into a real-time continuous biosensor. The developed signal processing architecture provides a structured way to perform parallel computations of data acquisition, data analysis, and the generation of a response signal that relates to the analyte concentration. The block size is a key parameter that allows one to control the trade-off between analytical precision and time delay. High-throughput signal analysis was demonstrated for a BPM sensor with 10,000 individual particles. We foresee that the signal processing architecture is also applicable to the other single-molecule sensing methods listed in Table 1, because all methods produce signal time series with discrete states, and in all methods, the concentration of analyte molecules affects the time characteristics of the signals. Therefore, the described signal processing architecture may also enable the development of other single-molecule methods into real-time continuous biosensors.

## Conclusions

We have developed a signal processing architecture for real-time continuous biosensing based on high-throughput single-molecule sensors. The signal processing architecture provides a structured way to perform parallel computation of data acquisition, data analysis, and generation of the response signal that relates to the analyte concentration, where the block size is a key parameter that controls the trade-off between analytical precision and time delay.

The signal processing architecture was tested on the BPM single-molecule sensing method and included all data analysis steps, including particle identification, particle tracking, drift correction, and the detection of state transitions in particle position time traces. The real-time analysis was validated on simulated data as well as experimental data of a competitive cortisol biosensor with more than 10,000 particles. The results show how the real-time signal analysis allows one to control the trade-off between measurement precision and signal processing time delay, indicating that an analytical precision of 10% and a signal processing time delay of 10 s can be achieved with a cortisol BPM sensor consisting of 10,000 particles. The implementation of the signal processing is computationally efficient and runs on a standard laptop, making it compatible with future wireless and wearable applications.

The assay that was used in this paper to study the real-time signal processing architecture is a competition assay with sensitivity in the low micromolar range. Without modification, the real-time signal processing architecture can be applied to sandwich assays in order to measure lower biomarker concentrations, such as BPM sandwich assays for protein and DNA detection that operate in nanomolar and picomolar concentration ranges.^21^

Furthermore, the signal processing architecture is also applicable to single-molecule sensing methods beyond BPM, e.g., based on fluorescence, plasmonics, or nanopores. Similar to BPM, these methods produce signal time traces with discrete states, and the time characteristics of the signals relate to the concentration of analyte molecules in solution. Therefore, the signal processing architecture developed in this paper can be broadly applied to single-molecule sensing methods, and we foresee that it can enable the development of a novel family of real-time continuous biosensors.

## Supporting Information Available

Particle identification methods, drift correction methods, simulations, quantitative evaluations, implementation of MM-CPD, filtering of activity under flow, and derivation of CV_C_ (PDF)

## Notes

The data and software of this study are available from the corresponding author upon reasonable request.
