# Supplementary material for: High-Throughput Single-Molecule Sensors: How Can the Signals Be Analyzed in Real Time for Achieving Real-Time Continuous Biosensing?
Source: ACS Sens. 2023 May 22;8(6):2271–81. doi: 10.1021/acssensors.3c00245 (PMC10294250; doi:10.1021/acssensors.3c00245)
Supplement: Supplementary file 1 — se3c00245_si_001.pdf [file se3c00245_si_001.pdf]

Supporting Information

High-throughput single-molecule sensors: how can the signals be analyzed in real time for achieving real-time continuous biosensing?

Max H. Bergkamp[1,3], Sebastian Cajigas[4], Leo J. van IJzendoorn[2,3], Menno W.J. Prins[1,2,3,4*]

[1]Department of Biomedical Engineering, Eindhoven University of Technology, 5612 AE Eindhoven, The Netherlands
[2]Department of Applied Physics and Science Education, Eindhoven University of Technology, 5612 AE Eindhoven, The Netherlands
[3]Institute for Complex Molecular Systems (ICMS), Eindhoven University of Technology, 5612 AE Eindhoven, The Netherlands
[4]Helia Biomonitoring, 5612 AR Eindhoven, The Netherlands
*Email: m.w.j.prins@tue.nl

S1. Particle identification

Particle identification is the process of defining a region of interest (ROI) around the particles that will be used for particle tracking. Particles that cannot be localized accurately, e.g., particles with too close neighbors, need to be filtered out. The particle identification methods presented here are specifically developed for identification of particles in images with a wide range of areal particle densities. Figure S1 shows the different steps and evaluation methods in the particle identification process for a typical brightfield image. These steps can be controlled with the parameters as listed in Table S1. This section includes a detailed description of all the steps in the particle identification procedure.

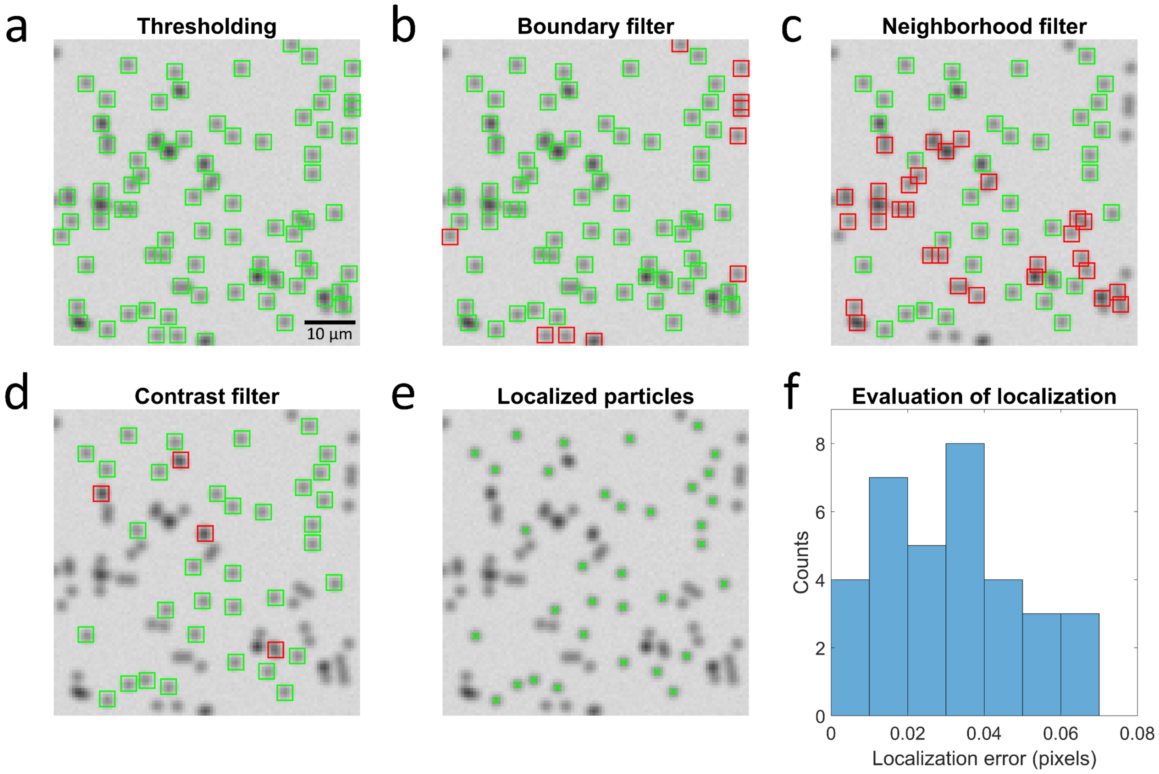

Figure S1: Methods and evaluation of particle identification demonstrated for a simulated zoomed-in image of 100x100 pixels (~25,000 particles/mm^2). (a) The green squares indicate the determined ROIs after thresholding. (b) The red squares indicate the ROIs that are discarded after applying the boundary filter. (c) The red squares indicate the ROIs that are discarded after applying the neighborhood filter. (d) The red squares indicate the ROIs that are discarded after applying the contrast filter. (e) The green crosses are the particle localizations that are computed by applying a localization algorithm to each ROI. (f) The particle localizations are evaluated by calculating the localization error of each localized particle, which is equal to the distance between the simulated and localized position.

Table S1: Default settings of particle identification.

Method	Parameter	Default value
Localization	ROI size	5 x 5 pixels ($ROI_{size,1D} = 5$)
Gaussian smoothing filter	σ_G	$ROI_{size,1D}/2$ pixels
Standard deviation filter	Neighborhood	$ROI_{size,1D}$ x $ROI_{size,1D}$ pixels
Thresholding	TH_I	4
Neighborhood filter	NF	1 pixel
Contrast filter	CF	1.3

S1.1 Thresholding

The first step in the particle identification process is setting a threshold based on the intensity. Ideally, a single threshold is defined automatically, for example based on the mean and standard deviation of the pixel intensities in the image. However, this approach results in a threshold that is dependent on the areal particle density in the image. In addition, this method is not robust for images with a nonhomogeneous background intensity. These problems can be avoided by setting a threshold based on the local intensity and the standard deviation of the intensity values in the background. The local intensity is determined by applying a Gaussian smoothing filter (with σ_G equal to $ROI_{size,1D}/2$) to the input image (see Figure S2b). Background pixels are defined as the pixels with an intensity value above the 90th percentile pixel intensity value in the Gaussian filtered image (see Figure S2d). Figure S2c shows the local standard deviation of the intensity values in the image, which is determined by applying a standard deviation filter to the image ($ROI_{size,1D}$ x $ROI_{size,1D}$ pixels neighborhood). The standard deviation of the background σ_{bg} is determined by averaging the local standard deviations of the defined background pixels. Finally, a threshold can be set based on the intensities of individual pixels in the input image I_{input} and the Gaussian filtered image I_{Gauss}:

$$I_{input} < I_{Gauss} - TH_I \cdot \sigma_{bg} \qquad \text{(S1)}$$

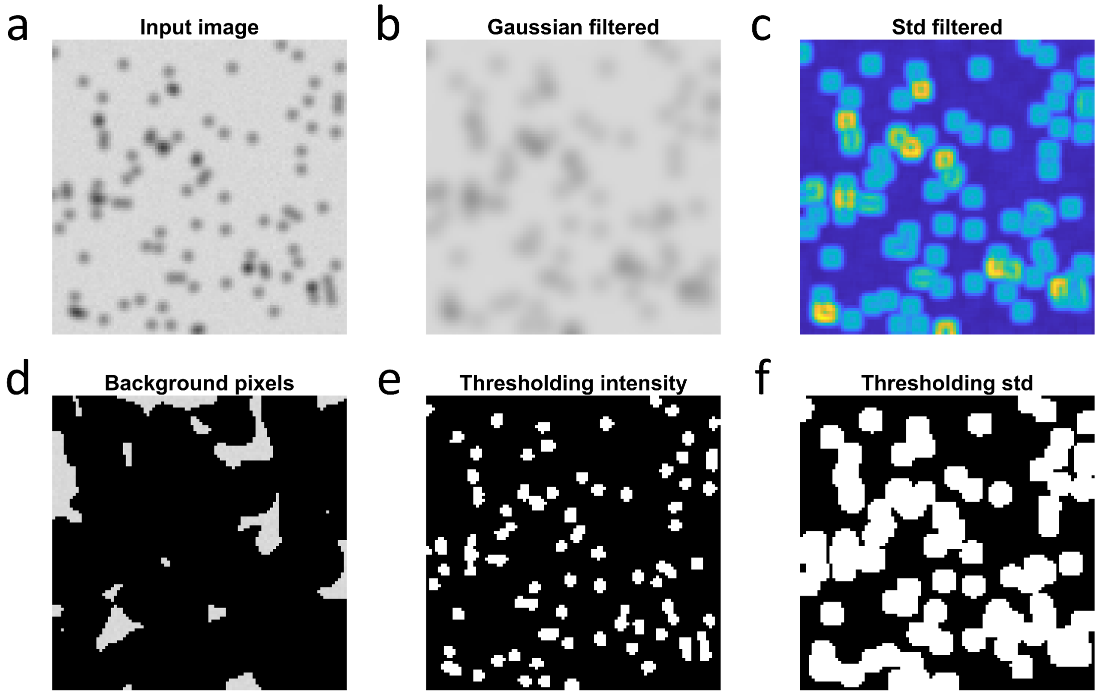

Figure S2: Thresholding methods. (a) Typical experimental zoomed-in brightfield image of particles (100x100 pixels). (b) Gaussian smoothing filter with standard deviation 2.5 pixels ($ROI_{size,1D}/2$). (c) Standard deviation filter with 5x5 pixels neighborhood. (d) Selected background pixels, which are the 10% pixels with the highest intensity in the Gaussian filtered image. (e) Binary image after thresholding based on local intensity. (f) Binary image after thresholding based on local standard deviation.

Figure S2e shows the binary image after thresholding with $TH_I = 4$. Additional to the intensity filter, a second thresholding method based on the local standard deviation is implemented to provide more robustness for background noise. Figure S2f shows the binary image after applying the local standard deviation threshold:

$$\sigma_{local} > TH_I \cdot \sigma_{bg} \quad \text{(S2)}$$

Figures S1a shows the ROIs that are defined around each pixel that is the local minimum in a 3x3 neighborhood and is equal to 1 in the product of the binary images in Figure S2e and S2f.

S1.2 Boundary filter

ROIs that are too close to the boundary are discarded (see Figure S1b). There should be at least NF pixels between the ROI and the boundary of the FOV, which is required to avoid errors in the neighborhood filter.

S1.3 Neighborhood filter

The neighborhood filter is applied to filter out particles with close neighbors (see Figure S1c). This filter discards ROIs if pixels in the local neighborhood of the ROI exceed the threshold based on intensity (see Figure S2e). The size of the local neighborhood is defined by the neighborhood filter parameter NF (see Figure S3). The neighborhood around the ROI can be enlarged to be more sensitive for filtering out particles with close neighbors. However, this reduces the total number of identified particles (see Figure S5b).

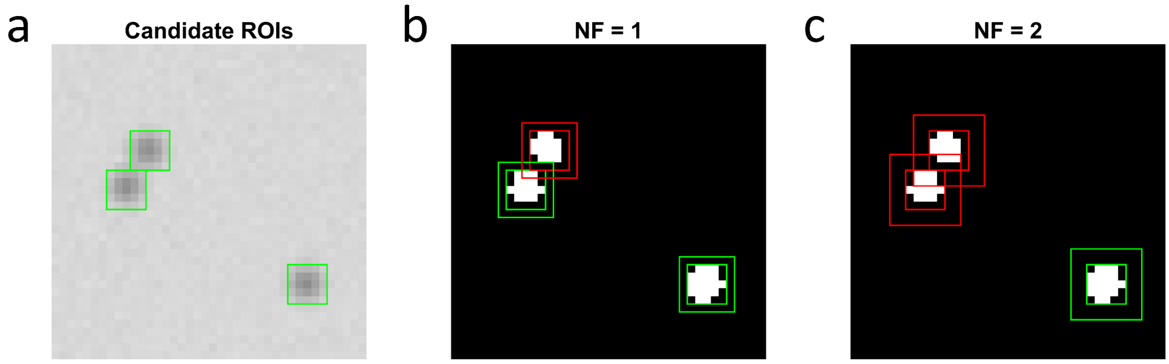

Figure S3: Neighborhood filter. (a) The green squares indicate the ROIs that are determined after thresholding. The neighborhood filter is applied to these ROIs to filter out particles with close neighbors. (b-c) The binary image is the image obtained after thresholding based on intensity (see Figure S2e). Each ROI is surrounded by two squares, the size of the inner square is equal to the ROI size. The distance between the inner and outer square is equal to NF. The ROIs surrounded by red squares are discarded, since there is at least one pixel in the region between the inner and outer square that exceeds the intensity threshold. The green squares are the ROIs that are accepted after applying the neighborhood filter.

S1.4 Contrast filter

The final filtering step is based on the contrast of the ROIs, i.e., the average intensity in the ROI I_{ROI} relative to the intensity of the background I_{bg}. For brightfield microscopy images the average intensity in the ROI around a particle is lower compared to the background intensity. The contrast of a ROI C_{ROI} in a brightfield microscopy image is defined as:

$$C_{ROI} = \frac{I_{bg} - I_{ROI}}{I_{bg}} \quad \text{(S3)}$$

I_{bg} is determined by taking the average of the $ROI_{size,1D}$ highest pixel intensity values in the local neighborhood around the ROI. The local neighborhood is simply an enlarged ROI around the candidate ROI (see Figure S3b).

Figure S4 shows the histograms of the C_{ROI} values in a simulated image and experimental image. A much larger spread is observed in the experimental image, which is likely due to fluctuations in the background intensity (see Figure S4c). The threshold for C_{ROI} is set based on a maximum factor CF that C_{ROI} is allowed to deviate from the median value of C_{ROI}. $CF = 1.3$ was found to be a good choice for both simulated and experimental data. Most of the ROIs that are discarded have a higher contrast, which is due to the presence of two particles in a single ROI. This contrast filter is robust for filtering out irregularities, e.g., a dust particle as illustrated in Figure S4c.

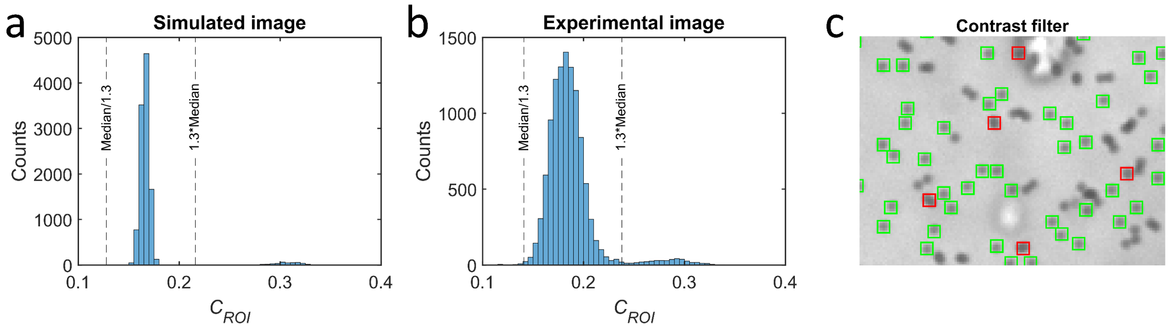

Figure S4: Contrast filter. (a) Histogram of C_{ROI} of 11,138 ROIs in a simulated image. The two dashed lines indicate the thresholds that are set (with $CF = 1.3$). (b) Histogram of C_{ROI} of 10,961 ROIs in an experimental image. The two dashed lines indicate the thresholds that are set. (c) Cropped experimental image (96x128 pixels) with the remaining ROIs after applying the contrast filter (green squares). The red squares indicate the ROIs that are discarded after applying the contrast filter.

S1.5 Evaluation of particle identification methods

The particle identification and localization can be evaluated by calculating the localization error of individual localizations, i.e., the distance between simulated and localized positions of the particles. A balance should be found between maximizing the number of identified particles and minimizing the localization errors. Figure S5 shows the localization error of the identified particles as a function of the nearest neighbor distance, after applying or leaving out different filtering steps. Figure S5a shows the results after applying all filtering steps as described in S1.1-S1.4 with the default settings as listed in Table S1. Clearly, the majority of the particles with a nearest neighbor distance smaller than 5 pixels ($ROI_{size,1D}$) are not identified. Some of the identified and localized particles with a nearest neighbor distance close to 5 pixels have an increased localization error. A more selective neighborhood filter ($NF = 2$) can be applied to reduce the average localization error (see Figure S5b). However, this is at the cost of the number of identified particles. Figure S5c and S5d show that not applying the neighborhood filter or contrast filter results in a significant fraction of localized particles with a large localization error.

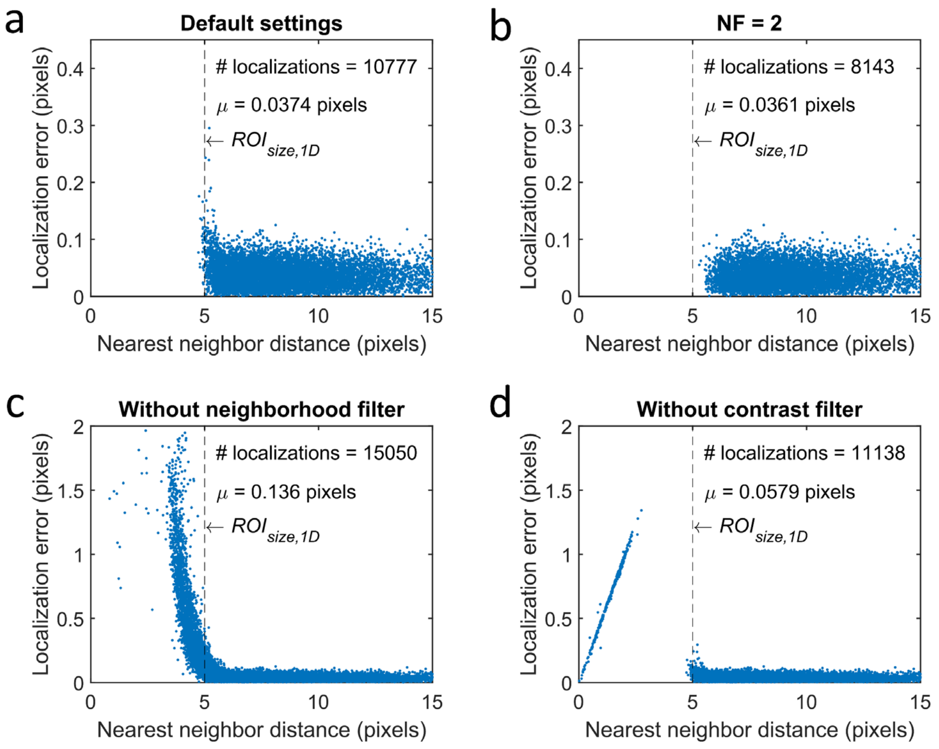

Figure S5: Effect of different filtering steps and settings on the identified particles and localization. Localization error as a function of the nearest neighbor distance of the localized particles. (a) Default settings. (b) More sensitive neighborhood parameter ($NF = 2$). (c) Without neighborhood filter. (d) Without contrast filter.

S1.6 Particle identification in experimental darkfield images

The particle identification methods are also suitable for particle identification in darkfield microscopy images. Figure S6 shows the results of the different particle identification steps applied to an experimental darkfield image. The default settings as listed in Table S1 were applied, and equations S1 and S3 were adapted for darkfield microscopy (see Table S2).

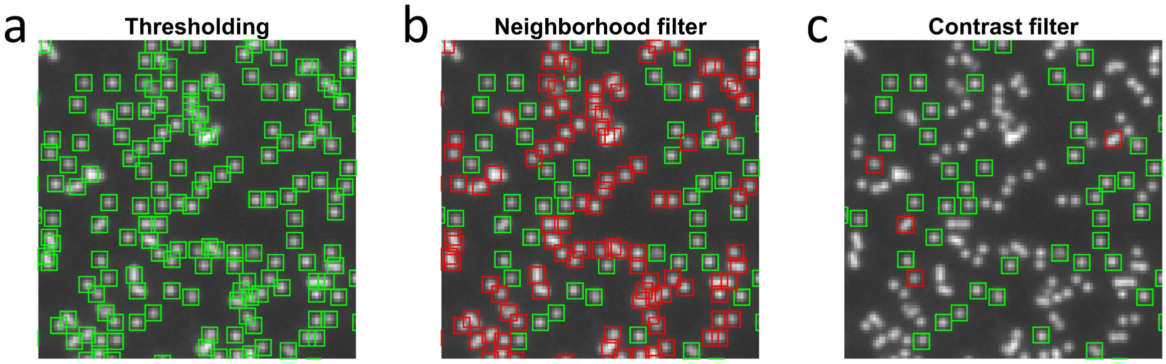

Figure S6: Particle identification in an experimental darkfield image (100x100 pixels) with a high areal particle density. (a) The green squares indicate the determined ROIs after thresholding. (b) The red squares indicate the ROIs that are discarded after applying the neighborhood filter. (c) The red squares indicate the ROIs that are discarded after applying the contrast filter.

Table S2: Equations for brightfield and darkfield microscopy images.

Method	Brightfield microscopy	Darkfield microscopy
Thresholding intensity	$I_{input} < I_{Gauss} - TH_I \cdot \sigma_{bg}$	$I_{input} > I_{Gauss} + TH_I \cdot \sigma_{bg}$
Contrast filter	$C_{ROI} = \frac{I_{bg} - I_{ROI}}{I_{bg}}$	$C_{ROI} = \frac{I_{ROI} - I_{bg}}{I_{bg}}$

S2. Drift Correction based on the Localization Cloud (DCLC)

Experimental artefacts can cause drift in the position of particles in consecutive images. The drift is determined by calculating the displacement of the center of mass of a subset of particles. For example, the drift estimation in x direction as a function of time $x_{DE}(t)$ determined from N_{DC} particles can be calculated from the x time traces of individual particles $x_i(t)$ as:

$$x_{DE}(t) = \frac{1}{N_{DC}} \sum_{i=1}^{N_{DC}} x_i(t) - x_i(0) \tag{S4}$$

Expression S4 can be used to calculate the drift between consecutive frames, resulting in a drift correction with a single-frame time resolution. Selecting a larger subset of particles can give a more precise estimation of the drift. However, the movement of individual particles should be considered, since it contributes to the estimated drift. For example, in a BPM sensor, the Brownian motion of the particles is limited by the tether according to confined diffusion[1,2].

The challenge is to find a subset of particles that gives the most precise estimation of the drift. Immobilized particles or particles with small movement are preferred. These particles can be already present in a BPM sensor, e.g., particles that are specifically or non-specifically bound for a long time or particles that are bound with multiple tethers. However, if only a limited number of particles with small movement is available, it might be beneficial to estimate the drift from a subset of particles that show larger movement, e.g., single-tethered particles.

Alternatively, immobilized particles can be added to the sample as fiducial markers, which is a common approach to facilitate drift correction[3,4]. However, this method would require an extra step in the sensor preparation protocol.

We therefore developed a generally applicable drift correction method: drift correction based on the localization cloud (DCLC), that automatically determines a subset of particles for calculating the drift. DCLC is a method that can be applied without requiring any input parameters. Here we demonstrate DCLC by analyzing a simulated dataset that includes drift and consists of 100 tethered particles and 10 immobilized particles. White Gaussian noise of 10 nm in both x and y direction was added to the simulated time traces to include localization uncertainty (see S3.1 for simulation details of the tethered particle time traces).

The first step in DCLC is performing an initial drift correction based on all particles (DCA) as shown in Figure S7a, the drift is calculated according to Equation S4 with the tethered and immobilized particles ($N_{DC} = N_{tethered} + N_{immobilized}$). The true drift correction error σ_{DC} of DCA is indicated, which can be determined by comparing the estimated drift in x and y direction, $x_{DE}(t)$ and $y_{DE}(t)$, to the simulated drift $x_{DS}(t)$ and $y_{DS}(t)$ in a time trace with T frames.

$$\sigma_{DC} = \sqrt{\frac{\sum_{t=1}^{T}(x_{DE}(t) - x_{DS}(t))^2 + (y_{DE}(t) - y_{DS}(t))^2}{T}} \tag{S5}$$

After this initial drift correction, it is possible to determine the properties of the particle motion patterns. The localization cloud is used as indication for the mobility of the particles, which can be calculated from the standard deviations in x and y direction (σ_x and σ_y). The localization cloud $\sigma_{lc,i}$ for particle i is defined as:

$$\sigma_{lc,i} = \sqrt{\sigma_{x,i}^2 + \sigma_{y,i}^2} \tag{S6}$$

Fig S7b shows the localization cloud for a tethered and immobilized particle before and after drift correction. The localization clouds of the individual particles are used to calculate the estimated drift correction error $\sigma_{DC,est}$ for the number of selected particles N_{DC} that are used to calculate the drift:

$$\sigma_{DC,est} = \frac{1}{N_{DC}} \sqrt{\sum_{i=1}^{N_{DC}} \sigma_{lc,i}{}^2} \tag{S7}$$

In DCLC, the N_{DC} particles with the smallest localization cloud are selected to calculate the drift, since the particles with the smallest localization cloud are assumed to provide the most precise indication of the drift. Therefore, the particles are sorted in ascending order based on the size of individual localization clouds. Figure S7c shows a histogram of the localization clouds, where the 10 immobilized particles with a small localization cloud can clearly be distinguished from the tethered particles.

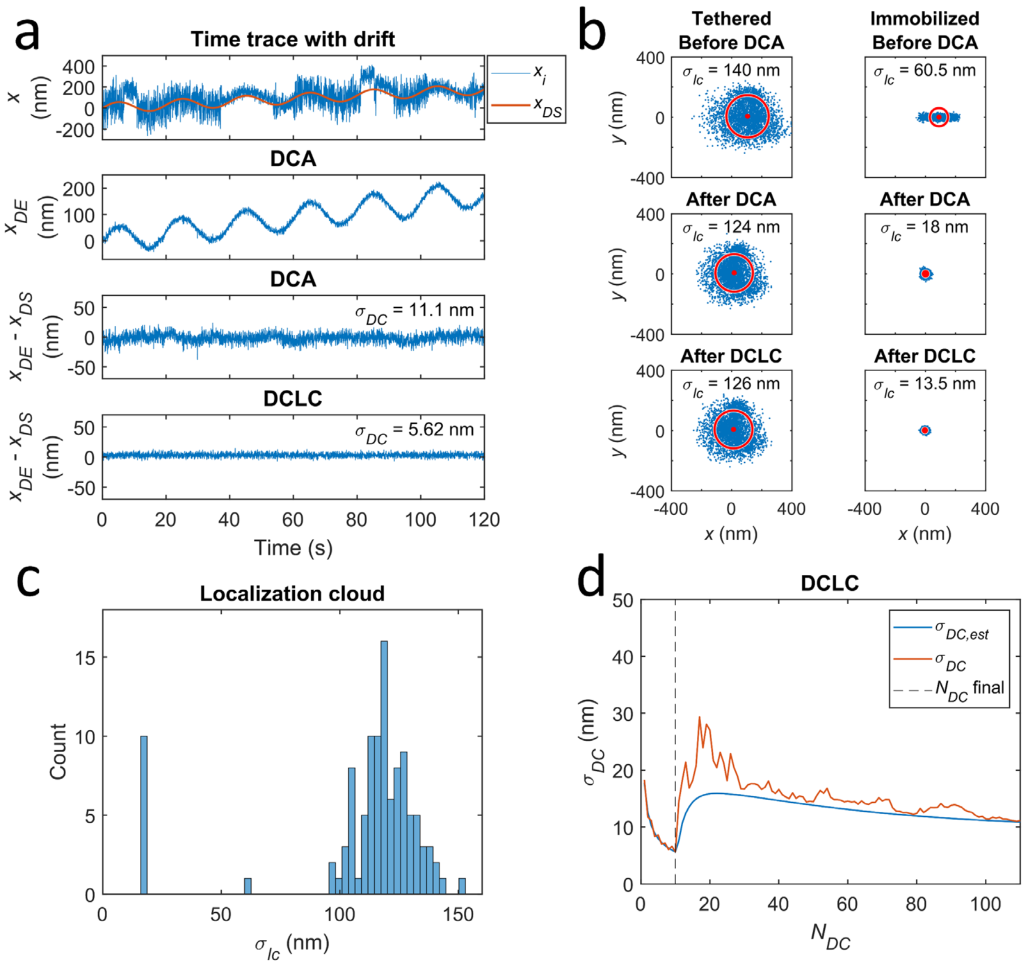

Figure S7: Drift correction based on the localization cloud (DCLC). (a) The top panel shows a simulated x time trace at 30 Hz with added drift. The simulated drift x_{DS} is a linear combination of sinusoidal drift (amplitude 50 nm, period 20 s) and linearly increasing drift. The second panel shows the estimated drift that was determined by calculating the displacement of the center of mass of all particles, referred to as DCA. The third and fourth panel show the difference between the simulated drift and the estimated drift with DCA and DCLC, respectively. The drift correction error is also indicated, showing that DCLC results in a more accurate drift correction compared to DCA. (b) Motion patterns and calculated localization clouds of a tethered and immobilized particle before and after applying DCA and DCLC. The red circles represent the localization clouds (radius is equal to σ_{lc}). (c) Histogram of the localization clouds of individual particles. (d) Estimated drift correction error $\sigma_{DC,est}$ and true drift correction error σ_{DC} as a function of the number of selected particles for calculating the drift N_{DC}. The smallest $\sigma_{DC,est}$ is reached for N_{DC}=10 (dashed vertical line) when only immobilized particles are selected for the drift correction.

Figure S7d shows the estimated drift correction error as a function of N_{DC}. The true drift correction error is also shown and follows the same trend as the estimated drift correction error. However, a large deviation between the true and estimated drift correction error is observed when a small number of tethered particles is included (10 < N_{DC} < 30). This deviation is attributed to the properties of the x and y distributions, which deviate from Gaussian distributions due to the presence of bound states (see top panel Figure S7a). When larger numbers of tethered particles are included, the deviation between the true and estimated drift correction error becomes smaller. This implies that the deviations caused by the presence of bound states are averaged out, which is expected because the positions of the bound states with respect to the center of the motion pattern are randomly generated. The subset of particles that gives the smallest estimated drift correction error is selected for the DCLC (dashed line). The bottom panel in Figure S7a shows that DCLC results in a smaller drift correction error compared to DCA.

Figure S8 shows the performance of DCLC as a function of the number of tethered particles with different numbers of immobilized particles in the dataset. These measurements were performed on a dataset consisting of 10,000 simulated tethered particle time traces of 2 minutes at 30 Hz (similar to the time trace in the top panel in Figure S7a). In samples without immobilized particles ($N_{immobilized} = 0$), the number of selected particles for calculating the drift was found to be equal to the number of tethered particles. Here, the drift correction error is decreasing as a function of the number of tethered particles. In samples with immobilized particles, N_{DC} was found to be equal to $N_{immobilized}$ until $N_{tethered}$ is above a certain value where DCLC selects all the particles ($N_{DC} = N_{tethered} + N_{immobilized}$) for the drift correction. This value represents the number of tethered particles for which the estimated drift correction error of DCA equals the estimated drift correction error of a drift correction based on only the immobilized particles. The drift correction error is therefore constant until this value is reached, afterwards it follows the same trend as a sample without immobilized particles. In measurements with large numbers of tethered particles, adding immobilized particles as fiducial markers is not needed, since performing a drift correction with only tethered particles already gives an accurate drift correction, e.g., the drift correction error is below 2 nm in a sample with 10,000 tethered particles. In this case, the drift correction error is well below the typical localization error of ~10 nm.

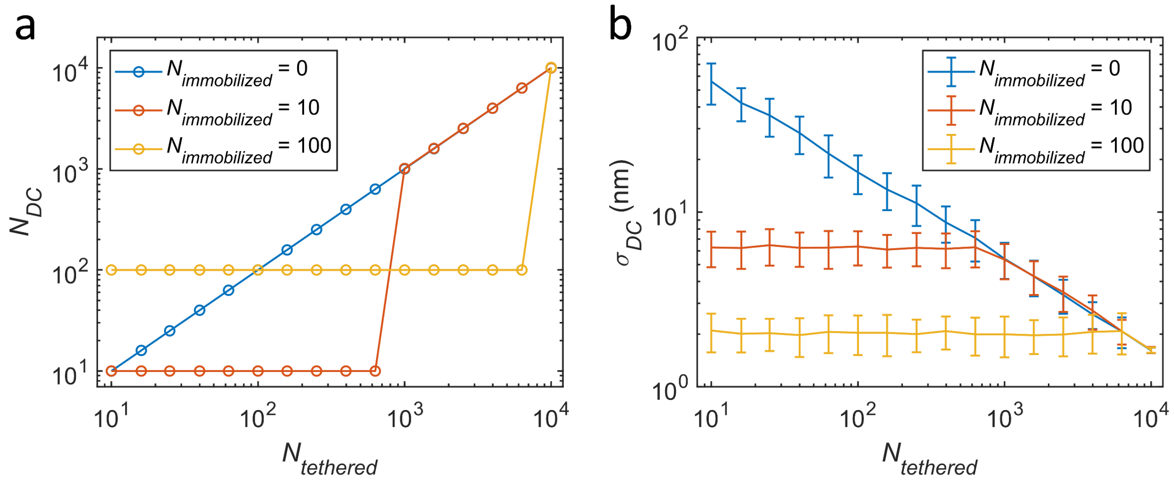

Figure S8: DCLC performance for different numbers of tethered and immobilized particles in a simulated dataset. (a) Number of selected particles for calculating the drift as a function of the number of tethered particles in the dataset. The colors indicate different numbers of immobilized particles. (b) Drift correction error as a function of the number of tethered particles in the sample. The error bars indicate the standard deviations between the obtained results from multiple simulations, in each simulation white Gaussian noise was generated and a random sample of $N_{tethered}$ particles was selected from the dataset.

S3. Simulations

Analysis of simulated data is needed for validation and quantitative evaluation of the performance of the signal processing architecture. Here, the simulation of a sequence of frames is described, for evaluating the particle tracking, drift correction and change point detection. Figure S9 shows how a sequence of frames can be simulated with particle time traces as input. Evaluating the particle identification and localization can be performed by simulating only a single frame. Section 3.1 explains how the x and y time traces of individual particles are simulated. Section 3.2 shows how a radial intensity profile can be extracted from experimental images and how it is used to simulate single frames. The described simulations were developed to provide a close match between simulated and experimental data.

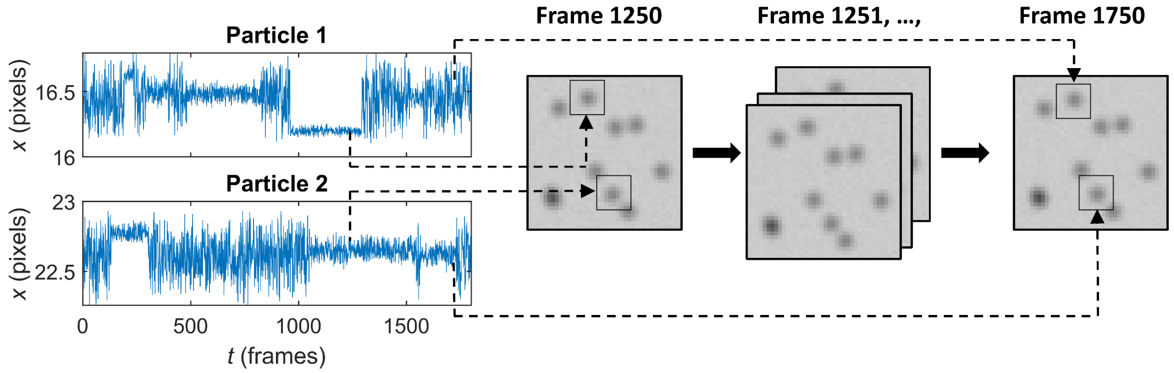

Figure S9: Generating a sequence of frames according to simulated time traces of individual particles. The sub-pixel resolution center of each individual particle in each frame corresponds to the position in the simulated x and y time traces.

3.1 Simulation of time traces

Individual x and y time traces of BPM particles are simulated according to the methods described by Bergkamp *et al.*[5]. The particle time traces include the parameters according to a typical BPM experiment, including the framerate (30 Hz), diffusion coefficient of a particle with a radius of 500 nm in water at room temperature ($D = 4.4 \cdot 10^{-13}\ m^2 s^{-1}$), state lifetimes (mean state lifetime 10 s, single-exponentially distributed) and multiple heterogeneous state position distributions (unbound state radius of 250 nm, bound states are randomly generated).

The simulated time traces are converted from nm to pixel units, according to the experimental pixel size. For each particle, a sub-pixel starting position is randomly generated in the FOV. There are no restrictions for the minimal distance between simulated particles.

3.2 Simulation of a single frame with multiple particles

Figure S10 explains the simulation of a single frame with multiple particles from a radial intensity profile. The radial intensity profile is extracted from an experimental image (see Figure S10a). High-resolution noise free images of each individual particle with a sub-pixel resolution center are added to the simulated image consecutively (see Figure S10b). The background and the noise free images of the particle are generated according to the radial intensity profile. Since there are no restrictions for the minimal distance between particles, it is possible that particles are simulated on approximately the same position (see addition of particle 10 in Figure S10b). After addition of all the particles, Poisson noise is added to the simulated image. The Poisson noise is added to a converted image with the number of photoelectrons per pixel. A conversion factor, equal to the number of photoelectrons per intensity count, is needed to generate this image. The conversion factor can be estimated from the gain of the camera. Finally, the image is converted back to obtain a simulated image with intensity counts.

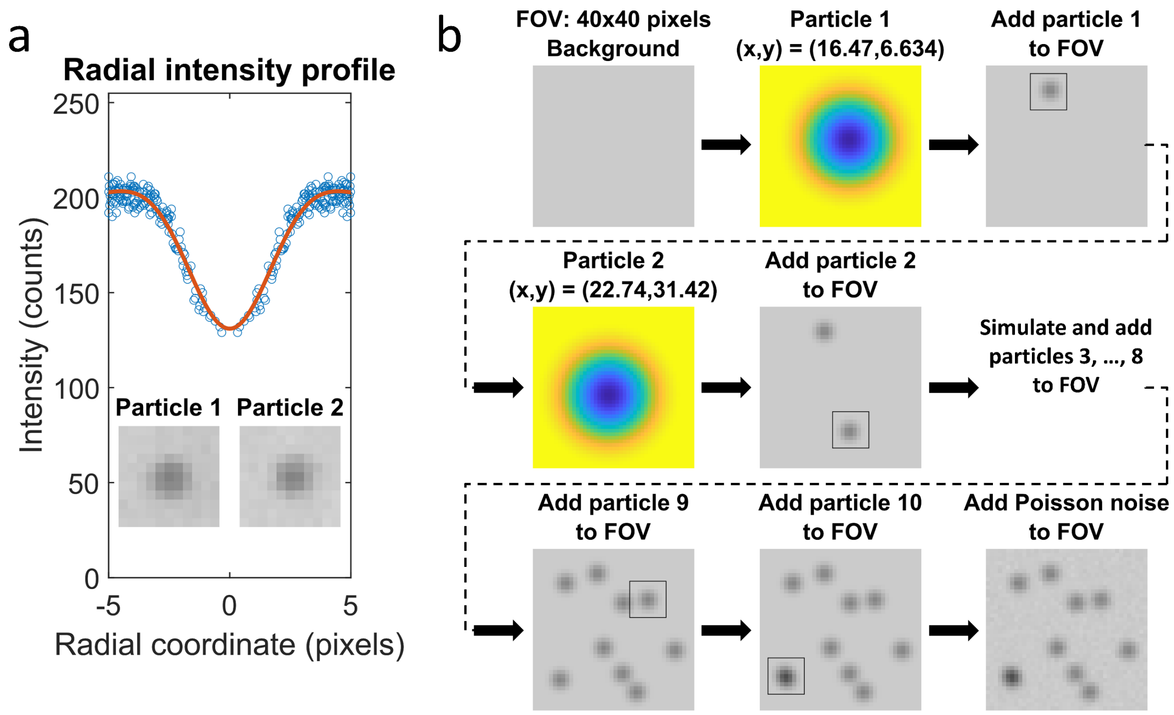

Figure S10: Simulation of a single image with multiple particles that is similar to an experimental image. (a) Extraction of a radial intensity profile from experimental images. The inset shows two experimental ROIs around particles that are used for determining the radial intensity profile. For each pixel in this ROI the radial coordinate is calculated, which is the distance between the center of the pixel and the localized center of the particle. The pixel intensities are plotted as a function of the radial coordinate (blue circles). The radial intensity profile is obtained by fitting a sum of four sines through the data. (b) Simulation of a single image with multiple particles. The first step is to simulate a FOV with the same background intensity as in the radial intensity profile. Particles are added to this image consecutively. For each particle, a high-resolution ROI is simulated based on the radial intensity profile and the sub-pixel resolution center. The pixel size in this high-resolution image is 5 times smaller in both dimensions. These high-resolution ROIs (45x45 pixels) are converted to ROIs of 9x9 pixels and added to the FOV. The final step is to add Poisson noise to the FOV, according to the number of photoelectrons per intensity count in the experimental image from which the radial intensity profile is extracted.

S4. Quantitative evaluation of particle tracking and drift correction

Particle tracking (PT) and drift correction (DC) can be evaluated by comparing the detected time traces to the simulated time traces. A sequence of 1800 frames at a framerate of 30 Hz with 20,000 tethered particles was simulated according to the methods described in the previous section. Additionally, a combination of linear and sinusoidal drift was added to the simulated time traces. This sequence of images was analyzed with the real-time software framework (RTSF). The only difference compared to real-time analysis of a biosensor is that the frames are now read from a local disk of a PC instead of capturing frames in real-time from the camera. Table S3 shows the simulation details and the number of analyzed particles in different steps after analyzing the simulated data with the RTSF. The number of tracked particles $N_{tracked}$ is slightly lower than the number of identified particles $N_{identified}$, which means that the tracking of some particles is discarded (see 2.2.5).

Table S3: Simulation settings and the number of particles that are in the different analysis steps after analyzing the simulated data with the RTSF.

FOV	# frames	$N_{simulated}$	$N_{identified}$	$N_{tracked}$	N_{DC}
2048x1536 pixels	1800	20,000	11,300	11,297	11,267

Figure S11 shows a comparison between the simulated and detected time traces. For evaluation of only the particle tracking, the simulated time traces that include drift should be compared with the output time traces without a drift correction (see Figure S11a). Drift correction can be evaluated by comparing the simulated drift to the estimated drift by the RTSF (see Figure S11b). The particle tracking error of an individual particle $\sigma_{i,PT}$ and drift correction error σ_{DC} are obtained by calculating the root mean squared error of the differences in the x and y time traces (see Equation S5). The drift correction error (~0.004 pixels) is approximately one order of magnitude smaller than the particle tracking error of this individual particle (~0.04 pixels).

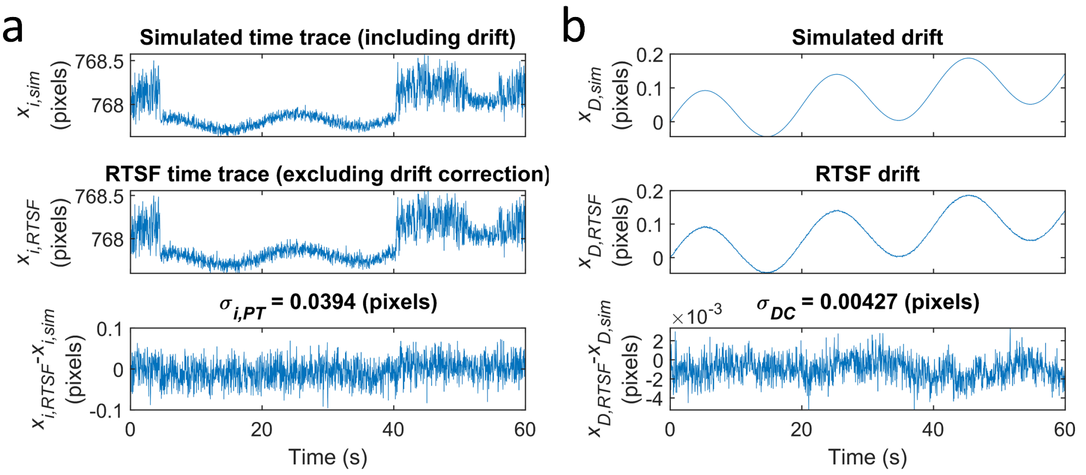

Figure S11: Evaluation of particle tracking and drift correction in the real-time software framework (RTSF). (a) Evaluation of the particle tracking. Comparison of the simulated time trace that includes drift to the detected time trace without a drift correction. (b) Evaluation of the drift correction. Comparison of the simulated drift with the estimated drift by the RTSF.

Figure S12 shows the overall error in the obtained time traces, which is a combination of errors in the particle tracking and the drift correction. Here, the error is dominated by the error in the particle tracking. The drift correction error has negligible influence on the combined error, indicating that an accurate drift correction error can be achieved with only tethered particles and addition of fiducial markers is not needed. Figure S12b shows the histogram of the errors of all individual time traces, where more than 99% of the time traces shows a combined error below 0.15 pixels. Furthermore, the combined error was above 1 pixel for only 3 out of 11,297 time traces (~0.03%). These results prove that the developed RTSF can track more than 10,000 particles with sub-pixel resolution.

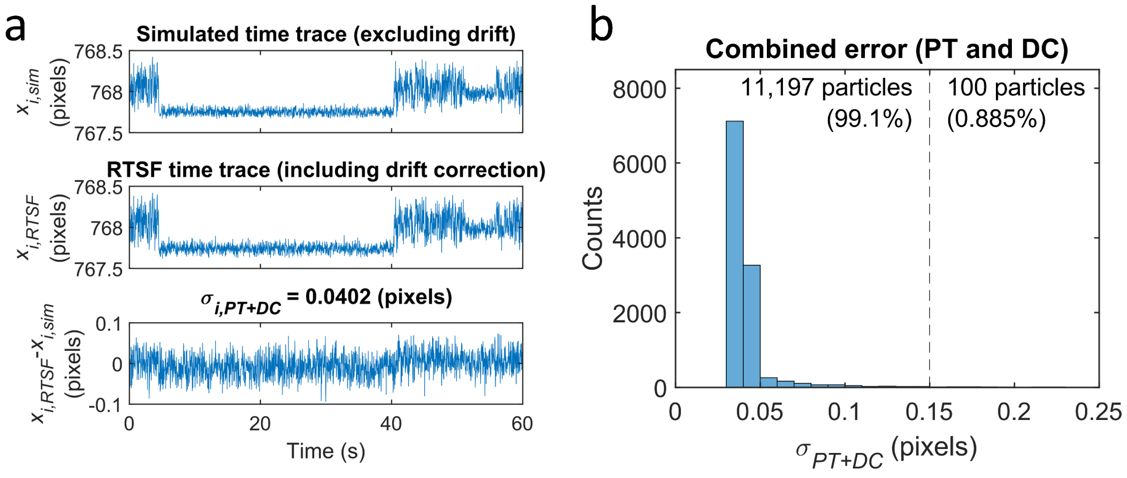

Figure S12: Combined error of particle tracking and drift correction. (a) Evaluation of the particle racking and drift correction. Comparison of the simulated time trace without drift to the detected time trace that includes a drift correction. (b) Histogram of the combined particle tracking and drift correction error of all individual particle time traces.

S5. Implementation and evaluation of change point detection

S5.1 Overlapping segments

Figure S13 explains why overlapping segments are needed for reliable change point detection close to the boundaries of a time trace. The need for overlap is caused by the fact that the MM-CPD algorithm compares distributions in neighboring windows for detecting change points. The MM-CPD algorithm relies on computing response time traces for different window sizes. These response time traces relate to the probability of a change point and a threshold can be applied to determine the change points. The response can only be calculated for data points with a distance to the boundary of the time trace that is larger than the window size. Therefore, change points are missed if the distance to the boundary is smaller than the window size. It should also be considered that change points might not be detected if the distance to the boundary is slightly larger than the window size. Figure S13 illustrates the change point detection for a window size of 40 frames. In this example, the change point is missed if it is located at frame 60 and detected if it is located at frame 80. If the change point is located at frame 60, the peak corresponding to the change point is not complete and is therefore not detected by the MM-CPD algorithm.

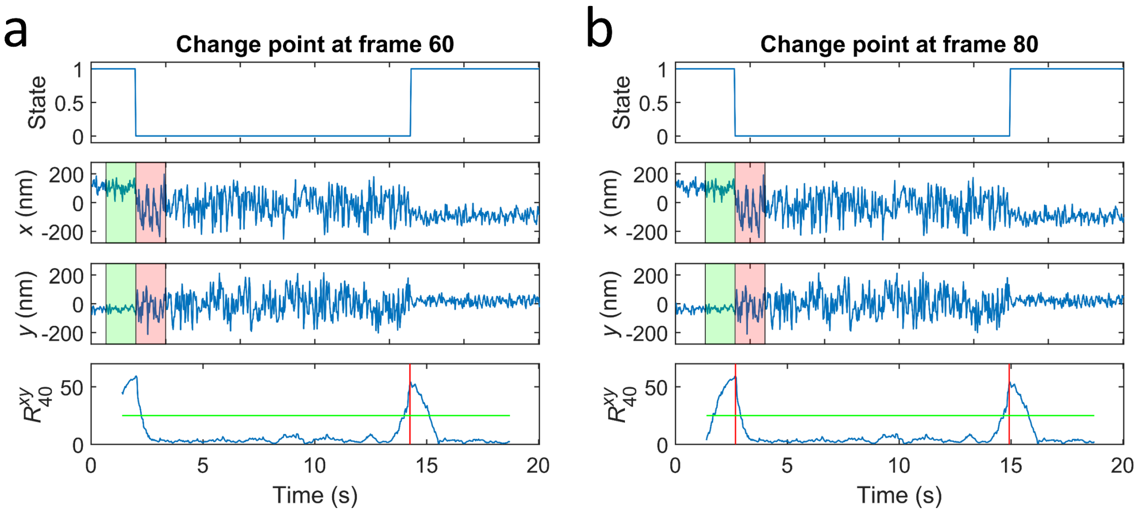

Figure S13: Change point detection near the boundary of a time trace. The top 3 panels in (a) and (b) show the simulated state, x and y time traces, respectively. In (a), there is a change point located at frame 60. The time trace shown in (a) is time-shifted in (b), with the change point located at frame 80. The green and red rectangles indicate the neighboring windows (window size of 40 frames) that are compared in the MM-CPD algorithm. The bottom panels show the response time trace, where the peaks correspond to the locations of the change points. The detected change points (red vertical lines) can be found by applying a threshold (green horizontal line). In (a), the peak corresponding to the change point at frame 60 is not complete, i.e., it intercepts the threshold only once. Therefore, the change point is not detected by the MM-CPD algorithm. In (b), the peak corresponding to the change point at frame 80 is complete and is therefore detected.

The size of the overlapping segments t_{OS} should be chosen such that reliable change point detection near the boundaries of a time trace is achieved. Here, this is investigated by evaluating whether the change point detection results are constant as a function of the block size t_{block}. For smaller block sizes, the average distance from a change point to the boundary of the time trace is smaller. Therefore, the effect of implementing overlapping segments on the change point detection results is expected to be more significant for smaller block sizes. Figure S14 shows the activity as a function of the block size for different sizes of the overlapping segments. These results were obtained by analyzing the simulated dataset from S4 with the real-time software framework. Figure S14a and S14b show that the size of the overlapping segments should be equal to two times the size of the largest window w_N in the MM-CPD algorithm w_N. By implementing $t_{OS} = 2 \cdot w_N$, the activity is constant as a function of the block size and only depends on the MM-CPD parameters.

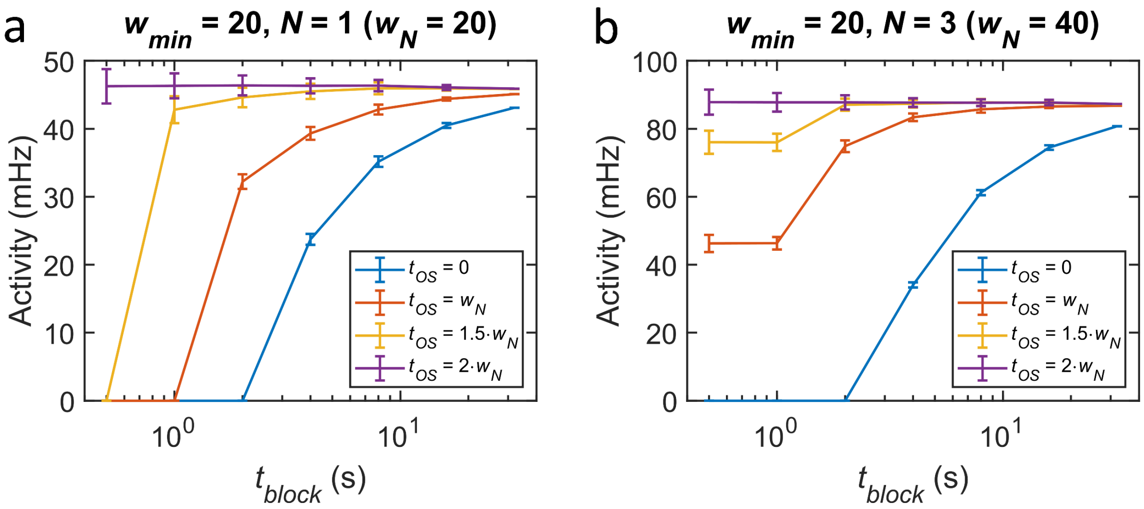

Figure S14: Activity as a function of the block size for different sizes of overlapping segments t_{OS}. The error bars indicate the standard deviation of the computed activities in multiple blocks. (a) MM-CPD settings: w_{min} = 20 frames and N = 1, which gives a largest window size w_N that is equal to 20 frames or 0.67 s (framerate 30 Hz). (b) MM-CPD settings: w_{min} = 20 frames and N = 3, which gives w_N = 40 frames.

In MM-CPD the results from multiple window sizes are combined to provide reliable change point detection for a wide range of state properties. Table S4 shows the required size of the overlapping segments dependent on the MM-CPD parameters.

Table S4: Influence of MM-CPD parameters on the required size of the overlapping segments.

w_{min} (frames)	N	w_{1-N}	w_N (frames)	t_{OS} (frames)
20	1	[20]	20	40
20	5	[20, 28, …, 80]	80	160
20	9	[20, 28, …, 320]	320	640

S5.2 Evaluation of change point detection

The CPD performance is evaluated by classifying the detected change points and missed change points. Detected change points can be either true positives (TP) or false positives (FP). Missed change points are classified as false negatives (FN). The F1-score is the evaluation parameter for the CPD performance and can have a maximum value of 1 if there are no false positives and no false negatives.

$$\text{F1-score} = \frac{TP}{TP + \frac{1}{2}(FP + FN)} \tag{S8}$$

It is interesting to study the influence of the block size and the MM-CPD parameters on the computational efficiency and the performance of the change point detection. Figure S15a shows the elapsed time, i.e., the total analysis time by the RTSF, as a function of the block size for different numbers of windows in the MM-CPD algorithm. The length of the analyzed time traces (obtained from the simulated dataset of S4) is 32 seconds excluding the overlapping segments. It is clearly visible, that the elapsed time significantly increases for small block sizes. The increase of analysis time is caused by an increase in the relative size of the overlapping segments with respect to the block size. The increase is larger when a larger number of windows is used, which is due to the increased size of the overlapping segments (see Table S4). A slight increase in elapsed time is observed for the largest block size, where the data is analyzed using a single block. The slight increase in elapsed time is attributed to a decrease in parallelization of computation processes when the number of blocks is decreased. Figure S15b

shows that increasing the number of windows can result in a more accurate change point detection. The F1-score is independent of the block size, which indicates that the overlapping segments are correctly implemented. N = 3 and N = 5 give approximately the same F1-score for this simulated dataset. Therefore, N = 3 would be the optimal choice for this simulated dataset. However, a larger number of windows might give a more accurate change point detection in experimental data, which can have larger heterogeneities in the time traces.

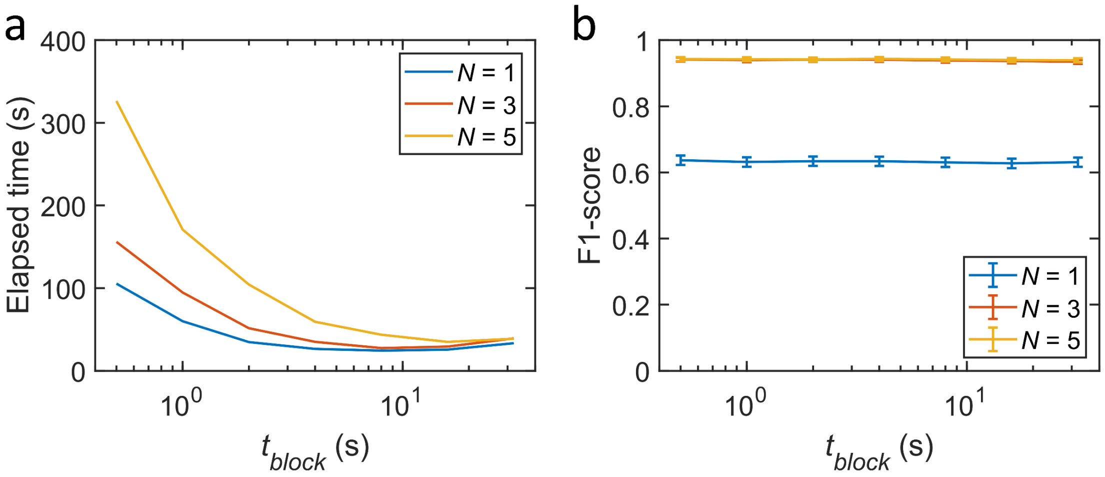

Figure S15: Evaluation of change point detection as a function of the block size for different numbers of windows N in the MM-CPD algorithm (w_{min} = 20 and threshold = 25). (a) Elapsed time as a function of the block size. (b) F1-score as a function of the block size. The error bars indicate the 95% confidence intervals calculated with the Clopper-Pearson method.

The experiments in this research were analyzed with w_{min} = 20, N = 5 and a threshold of 25, which gives accurate change point detection on simulated data (see Figure S15b) and excellent computational performance for block sizes of 1 minute. Decreasing the block size was not needed in this research, since the timescales of physical and chemical processes in the BPM sensor are typically a few minutes. In experiments where the timescales are shorter, a smaller block size is desired. In that case the choice of the windows in the MM-CPD algorithm should be carefully selected to avoid high CPU usage and long computational time delays.

S6. Filtered activity in BPM measurements under flow

In BPM measurements performed under flow, outliers were observed in the computed activity values. Figure S16a shows the activity values as a function of time within a single measurement block of 1 minute. At around 25 s a spike is observed in the activity, causing deviations in the average activity in that specific block. In the time trace of the computed drift, a clear step was observed at the time of the spike in the activity (see Figure S16b). The observed spikes in the activity signal most likely relate to instabilities of pressure and flow in the system, potentially caused by the pump, by valves, by air bubbles, or by other system components.

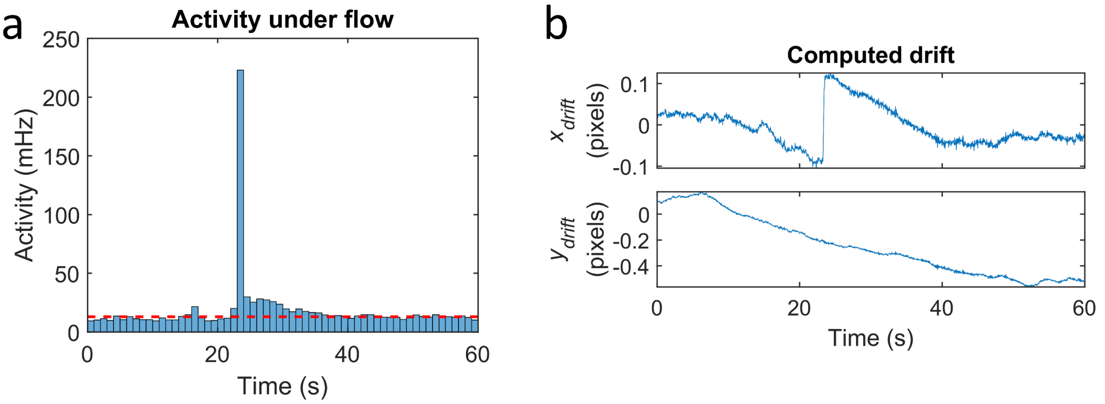

Figure S16: BPM activity measurements under flow. (a) Histogram of the activity as a function of time with a bin width of 1 second for a measurement block of 1 minute. The red horizontal dashed line indicates the median value. Activity values below the median are used for calculating the filtered activity in a single block of 1 minute. (b) Computed x and y drift time traces corresponding to the data in (a).

Figure S17 shows the activity values as a function of time with and without applying a filter for outliers. The filtered activity was obtained by taking the average of the activity values below the median value (indicated by the red dashed line in Figure S16a). The filtered activity is always lower than the activity without a filter, because all above-median values including the spikes are excluded. Large differences between filtered and unfiltered activity are observed between 100 and 115 minutes; here the flow was repeatedly unstable, as was also visible in the drift correction (not shown). The results of Fig. S17 show that a median-based filter method is suited for removing spike artefacts in the activity that can appear when BPM measurements are performed under flow.

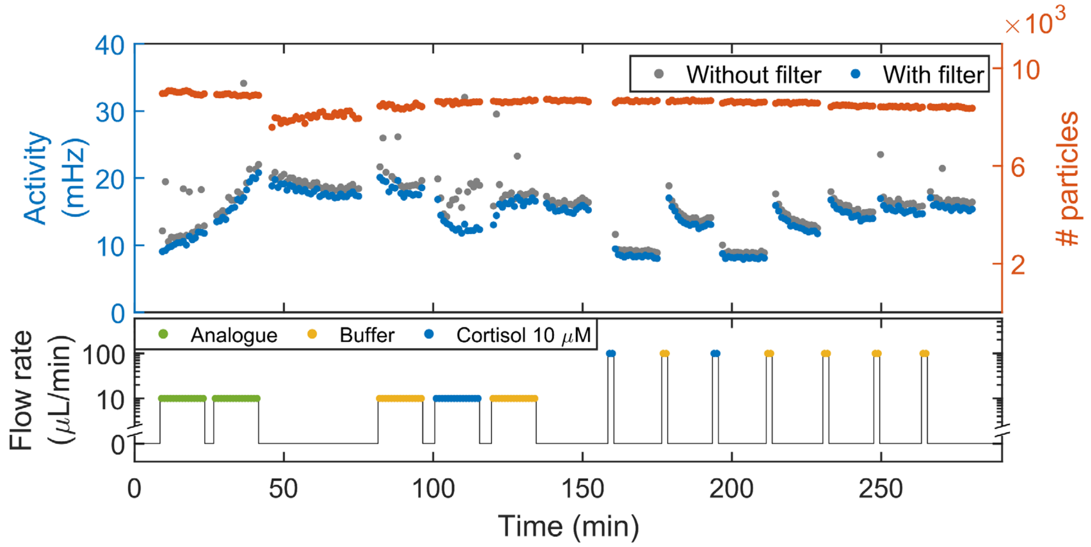

Figure S17: The top panel shows the filtered activity (blue, left axis), the activity before applying the filter (gray, left axis) and the number of particles (red, right axis) as a function of time. The bottom panel shows the flow protocol as a function of time and indicates the different solutions: analogue (green), buffer (yellow) and 10 µM cortisol (blue), that are flowed in.

S7. Coefficient of variation of concentration determination

The dose-response curve was fitted with a sigmoidal curve. The signal S, i.e., the activity, as a function of the concentration C is given by:

$$S = S_{min} + \frac{S_{max} - S_{min}}{1 + \frac{C}{EC_{50}}} \quad \text{(S9)}$$

The coefficient of variation of concentration determination CV_C is defined as the standard deviation of the measured concentration σ_C divided by the concentration C:

$$CV_C = \frac{\sigma_C}{C} \quad \text{(S10)}$$

The standard deviation of the concentration can be estimated from the standard deviation of the signal and σ_S and the absolute value of the slope of the dose-response curve:

$$\sigma_C = \frac{\sigma_S}{\left|\frac{dS}{dC}\right|} \quad \text{(S11)}$$

The derivative of the signal (Equation S9) with respect to the concentration is given by:

$$\frac{dS}{dC} = \frac{S_{min} - S_{max}}{EC_{50} \cdot (1 + \frac{C}{EC_{50}})^2} \quad \text{(S12)}$$

Since S_{max}>S_{min} and EC_{50}>0, the absolute value of S12 is given by:

$$\left|\frac{dS}{dC}\right| = \frac{S_{max} - S_{min}}{EC_{50} \cdot (1 + \frac{C}{EC_{50}})^2} \quad \text{(S13)}$$

Combining equations S10, S11 and S13 allows one to determine CV_C from the fitted curve and the standard deviation of the signal:

$$CV_C = \frac{\sigma_S \cdot EC_{50} \cdot (1 + \frac{C}{EC_{50}})^2}{C \cdot (S_{max} - S_{min})} \quad \text{(S14)}$$

S7.1 Allan deviation

The Allan deviation is used to determine the noise in the signal as a function of the averaging time. The Allan deviation allows one to distinguish between different noise sources present in the signal[6,7]. Typically, the Allan deviation decreases as a function of the averaging time for short timescales caused by averaging out the noise. For longer averaging times, the Allan deviation might increase due to the presence of amplitude fluctuations on long timescales, e.g., caused by physical processes in the system.

Here, the Allan deviation in the activity as a function of the averaging time, i.e., the block size in an experiment, is used as an estimator for the noise in the signal as a function of the block size. The σ_S in Equation S14 is replaced by the Allan deviation in the activity $ADEV_S$:

$$CV_C(t_{block}) = \frac{ADEV_S\,(t_{block}) \cdot EC_{50} \cdot \left(1 + \frac{C}{EC_{50}}\right)^2}{C \cdot (S_{max} - S_{min})} \quad \text{(S15)}$$

References:

(1) Manzo, C.; Garcia-Parajo, M. F. A Review of Progress in Single Particle Tracking: From Methods to Biophysical Insights. *Reports Prog. Phys.* **2015**, *78* (12), 124601. https://doi.org/10.1088/0034-4885/78/12/124601.

(2) Destainville, N.; Salomé, L. Quantification and Correction of Systematic Errors Due to Detector Time-Averaging in Single-Molecule Tracking Experiments. *Biophys. J.* **2006**, *90* (2). https://doi.org/10.1529/biophysj.105.075176.

(3) Ovesný, M.; Křížek, P.; Borkovec, J.; Švindrych, Z.; Hagen, G. M. ThunderSTORM: A Comprehensive ImageJ Plug-in for PALM and STORM Data Analysis and Super-Resolution Imaging. *Bioinformatics* **2014**, *30* (16). https://doi.org/10.1093/bioinformatics/btu202.

(4) Lee, S. H.; Baday, M.; Tjioe, M.; Simonson, P. D.; Zhang, R.; Cai, E.; Selvin, P. R. Using Fixed Fiduciary Markers for Stage Drift Correction. *Opt. Express* **2012**, *20* (11). https://doi.org/10.1364/oe.20.012177.

(5) Bergkamp, M. H.; Van IJzendoorn, L. J.; Prins, M. W. J. Real-Time Detection of State Transitions in Stochastic Signals from Biological Systems. *ACS Omega* **2021**, *6* (27). https://doi.org/10.1021/acsomega.1c02498.

(6) Czerwinski, F.; Richardson, A. C.; Oddershede, L. B. Quantifying Noise in Optical Tweezers by Allan Variance. *Opt. Express* **2009**, *17* (15). https://doi.org/10.1364/oe.17.013255.

(7) Huhle, A.; Klaue, D.; Brutzer, H.; Daldrop, P.; Joo, S.; Otto, O.; Keyser, U. F.; Seidel, R. Camera-Based Three-Dimensional Real-Time Particle Tracking at KHz Rates and Ångström Accuracy. *Nat. Commun.* **2015**, *6*. https://doi.org/10.1038/NCOMMS6885.
